# Plant Pyranocoumarins: Description, Biosynthesis, Application

**DOI:** 10.3390/plants11223135

**Published:** 2022-11-16

**Authors:** Maria T. Khandy, Anastasia K. Sofronova, Tatiana Y. Gorpenchenko, Nadezhda K. Chirikova

**Affiliations:** 1 Laboratory of Cell and Developmental Biology, Federal Scientific Center of East-Asia Terrestrial Biodiversity, Far Eastern Branch of the Russian Academy of Sciences, Stoletiya Vladivostoka Ave. 159, Vladivostok 690022, Russia; 2 Laboratory of Biomedical Cell Technologies of the Center for Genomic and Regenerative Medicine, Institute of Life Sciences and Biomedicine, Far Eastern Federal University, FEFU Campus, 10 Ajax Bay, Russky Island, Vladivostok 690922, Russia; 3 Department of Biology, Institute of Natural Sciences, M.K. Ammosov North-Eastern Federal University, 58 Belinsky Str., Yakutsk 677000, Russia

**Keywords:** pyranocoumarins, coumarins, biosynthesis of pyranocoumarins, classification of pyranocoumarins, structure of pyranocoumarins, functions of pyranocoumarins

## Abstract

This overview article contains information about pyranocoumarins over the last 55 years. The article is based on the authors’ phytochemical and physiological studies in vivo and in vitro as well as search and analysis of data in literature available on Google Scholar, Web of Science, PubMed, and ScienceDirect before January 2022. Pyranocoumarins are synthesized in plants of the *Apiaceae*, *Rutaceae* families, and one species in each of the *Cornaceae*, *Calophyllaceae*, and *Fabaceae* families can synthesize this class of compounds. The physiological role of these compounds in plants is not clear. It has been proven that these substances have a wide range of biological activities: anti-cancer, anti-spasmatic, and anticoagulant, and they also inhibit erythrocyte lysis and accumulation of triacylglycerides. The overview generalizes the modern understanding of the classification, structure, and biological activity of natural pyranocoumarins, and summarizes dispersed data into a unified scheme of biosynthesis. The review analyzes data on the localization and productivity of these substances in individual organs and the whole plant. It discusses a link between the unique structure of these substances and their biological activity, as well as new opportunities for pyranocoumarins in pharmacology. The article evaluates the potential of different plant species as producers of pyranocoumarins and considers the possibilities of cell cultures to obtain the end product.

## 1. Introduction

Throughout the entire written history of the humanity, and certainly long before that, plants have been used as medicines [1]. Even though knowledge of medical plants has been mainly obtained empirically, this resulted in the development of the main pharmaceutical drugs necessary for modern medicine. It has been calculated that almost half of the drugs approved over the last 30 years are of natural origin or are produced from natural compounds, many of which are plants [2]. Some famous examples of medicines include phenol compounds of natural origin: cholagogue flamen produced from *Helichrysum arenarium*, antimicrobial shikonin produced from *Lithospermum erythrorhizon*, anticoagulant dicoumarin produced from plants of the *Melilótus* genus, etc. A big part of plant chemical diversity remains unstudied, and there are more than 28,000 species of medical plants that may potentially be used to develop new drugs [3]. Searching for unique compounds having high biological activity is the basis of development of the global pharmacological industry. At the same time, three species of higher plants become extinct every year, which is 500 times faster than natural extinction [4]. Because of this, humanity irretrievably loses unique structures of natural compounds that may have biological activity and become a solution in treating certain diseases.

Pyranocoumarins are rare secondary metabolites of plants: coumarin containing a pyran core. Coumarins are widely spread compounds of plant origin that are essential for the life of plants and that perform protective and regulatory functions [5]. Depending on the structural modification, they have a number of physiological activities: anti-spasmodic action, capillary-reinforcing, sedative, diuretic, vermicidal, analgesic, and antimicrobial action. Some coumarins stimulate functions of the central nervous system, reduce the blood cholesterol, prevent clot formation in blood vessels and promote their dissolution, increase skin UV-sensitivity, accelerate ulcer healing, stimulate breathing, and increase the arterial pressure [6]. Unlike coumarins, the physiological role of pyranocoumarins in plants is not clear. However, there is data describing various biological activities of these secondary metabolites in animals and humans: anti-cancer, anti-spasmatic, and anticoagulant activities. Moreover, they inhibit erythrocyte lysis and accumulation of triacylglycerides.

Pyranocoumarins represent a narrow group of substances synthesized in a limited number of plant species of the *Apiaceae* and *Rutaceae* families, and rarely in other families. Mechanisms of pyranocoumarin synthesis in plant cells and in whole plants are poorly investigated.

Literature presents disparate data for the diversity of pyranocoumarins in flora and their biological activity in various test models. However, these data need to be systematized and aggregated in order to predetermine the areas of future studies. It is necessary to understand the structural diversity of pyranocoumarins and their functions since even small structural changes define physiological properties. Overviews of pyranocoumarins in the global scientific literature are rare. A very interesting overview by Beena and Sathya Pooja in 2022 was dedicated to the heterocycle of pyranocoumarins, namely to important analogs of pyranocoumarin with various biological activities [7].

Generalized experimental results of R&D centers of different countries analyzed in this paper allow us to recreate the complicated biosynthesis path of pyranocoumarins, show the important functional role of these substances, and find the conditions supporting synthesis of this narrow group of substances in various plant species. The article is based on our own phytochemical and physiological studies in vivo and in vitro, as well as a search and analysis of the literature available on Google Scholar, Web of Science, PubMed, and ScienceDirect before January 2022. Thus, this overview is intended to systematize and analyze the information in the available literature concerning this narrow and unique group of substances.

## 2. Pyranocoumarins Structure and Classification

**Figure 1:**
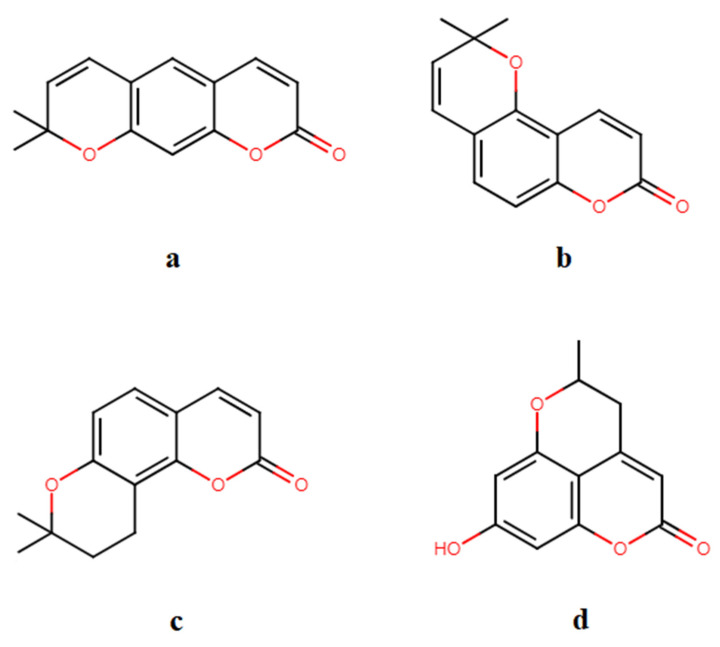
Natural groups of pyranocoumarins: (**a**) linear pyranocoumarin; (**b**) 5,6-angular pyranocoumarin; (**c**) 6,7-angular pyranocoumarin; (**d**) condensed pyranocoumarin.

Pyranocoumarins belong to the class of coumarins, derivatives of 2H-1-benzopyran-2-on [8]. These substances containing a pyran core condensed with coumarin at 4,5; 5,6; 6,7; 7,8 positions and having substitutes in pyran, benzene, and pyridine rings [9,10]. Structurally, depending on how the pyran ring is formed, there are three groups of pyranocoumarins: linear (Figure 1a), angular (Figure 1b,c), and condensed (Figure 1d). Natural linear pyranocoumarins have substitution in the parent benzenoid ring and/or in heteronuclear rings [11]. The structures of the pyranocoumarins overviewed here are represented in Supplementary Materials (Table S2 and Figure S1).

## 3. Biosynthesis of Pyranocoumarins in Cells

**Figure 2:**
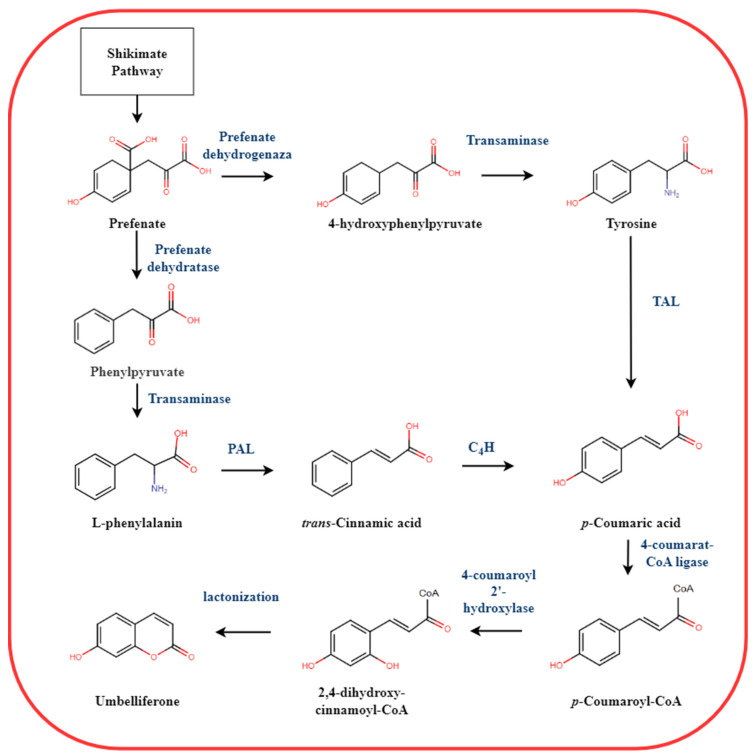
Biosynthesis pathway of umbelliferone, a precursor of pyranocoumarins.

A precursor of all pyranocoumarins is umbelliferone whose biosynthesis is implemented via the phenylpropanoid pathway (Figure 2) [12]. Exposed to phenylalanine ammonia-lyase (PAL)), L-phenylalanine transforms into trans-cinnamic acid followed by hydroxylation by cinnamate-4-hydroxylase (C4H), forming p-coumaric acid. This acid may also be formed from another product of the shikimate pathway—L-tyrosine, when exposed to tyrosine ammonia lyase (TAL). Exposed to 4-coumarat-CoA ligase, p-coumaric acid provides p-coumaroyl-CoA. Then the molecule is once again hydroxylated by cinnamate/coumarate-2-hydroxylase to produce 2,4-dihydroxy acid (umbellic acid) followed by rotation of the double bond neighboring the carbonic acid group. Finally, the intra-molecular attack of the hydroxyl group C2 on the carbonic acid group encloses the ring and forms the phenylpropanoid umbelliferone, precursor of pyranocoumarins [13].

**Figure 3:**
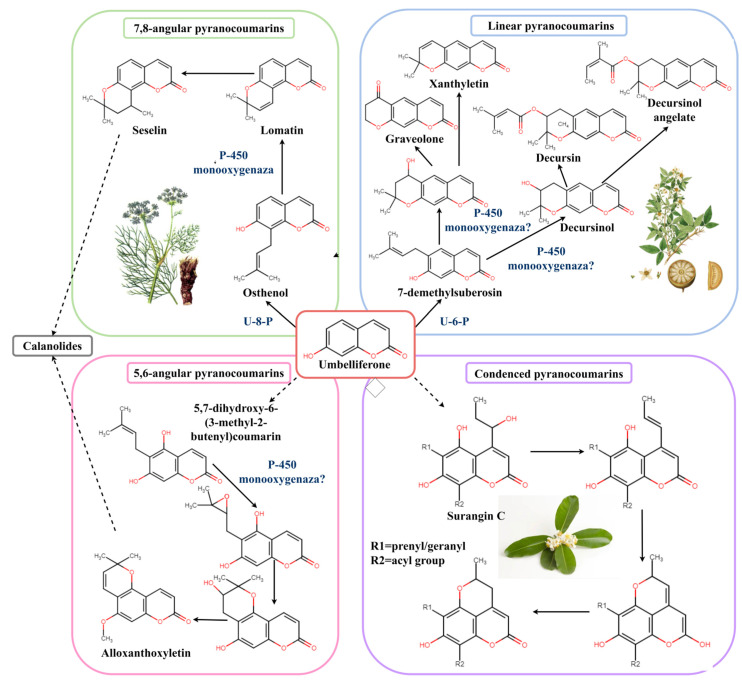
Biosynthesis of pyranocoumarins.

Exposed to prenyltransferases, umbelliferone forms either osthenol, a precursor of 7,8-angular pyranocoumarins, or 7-demethylsuberosin—a precursor of linear pyranocoumarins [14]. In an experiment with carbon label conducted for orange seedlings (*Citrus sinensis* (L.) Osbeck) and rangpur seedlings (*Citrus limonia* Osbeck), it has been proven that the coumarin fragment of pyranocoumarins xanthyletin and seselin originate from the shikimate pathway (L-phenylalanine), and the pyran ring is formed via the methyl–erythrite–phosphate pathway [15]. Transformation and coupling of substitutes are most likely related to various transferases. There are suggestive data on the formation of decursinol and its derivatives [12,14]. Zhao with coauthors combined methods of transcriptomics and metabolomics to build the biosynthesis pathway of coumarins in *Peucedanum praeruptorum* Dunn, including pyranocoumarins, and suggested that genes CYP450 and MDR participated in coumarin biosynthesis [16]. The data about the biosynthesis pathway of other two groups of pyranocoumarins is limited by what is shown in Figure 3. Gómez-Robledo with coauthors made structural analysis of calanolides (dipyranocoumarins) and transcriptomic analysis using RNA sequencing and suggested biosynthesis pathways and unigene candidates in the dataset of transcriptome potentially participating in biosynthesis pathways of umbelliferone and calanolide [13]. Biosynthesis pathways of condensed pyranocoumarins are suggested to be started with Sturangin C, a coumarin characteristic of the *Mammea* genus, which is the only genus of plants with condensed pyranocoumarins [17,18]. In any case, the precursor must have substitutes of 1-hydroxypropyl and prenyl or geranyl at C4 and C6 positions of coumarin (Figure 3). Therefore, the tandem of transcriptomic and biochemical analyses can give new opportunities in studying biosynthesis pathways of pyranocoumarins. Herewith, experiments may use producing plants without employing model objects.

## 4. Pyranocoumarins Distribution in Plant World

**Figure 4:**
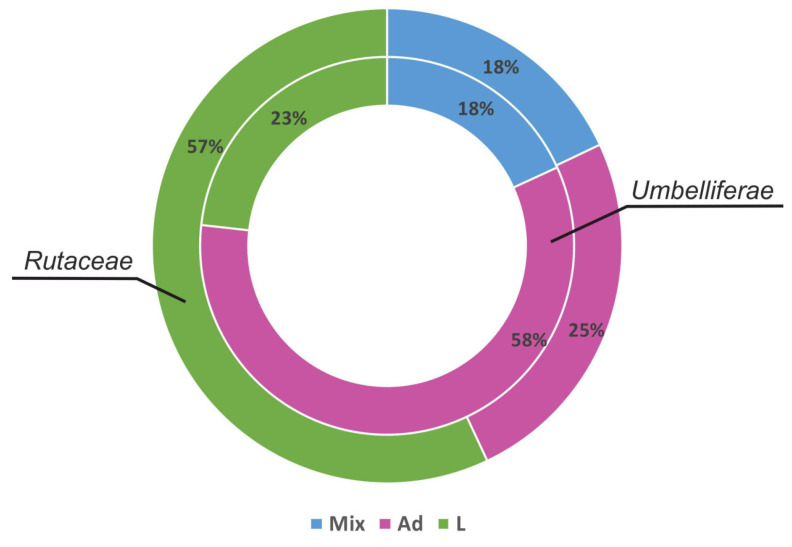
Distribution of linear and angular pyranocoumarins within the *Umbelliferae* and *Rutaceae* families. Mix-Ad and L pyranocoumarins; Ad-angular 7,8-pyranocoumarin; L-linear 6,7-pyranocoumarin.

Pyranocoumarins are found only among the *Umbelliferae* and *Rutaceae* families, and in one species from each of *Calophyllaceae*, *Cornaceae*, and *Leguminosae*. Distribution of pyranocoumarin forms within primary families producing these substances shows significant predomination of one of the structural forms within one family. For example, 58% of substances in the *Umbellíferae* family are represented by angular form and only 23% are characterized by linear form. The *Rutaceae* family have predominating linear form of structure in 57% of species. Interestingly, the number of species where substances with different molecular structure are found is 18% or less in both families (Figure 4). This distribution of pyranocoumarin forms may be due to the evolutionary early occurrence of species and existence of a pathway from 7-dimethylsuberoside, while the more evolutionary developed *Rutaceae* have osthenol derivatives as predominating. Groups of substances with the condensed form are described only in *Mammea siamensis* T. Anders. from the *Calophyllaceae* family. Distribution of 5,6-angular pyranocoumarins is also poorly studied. It should be noted that this may change both because of the differentiation and determination methods of the substances and the increased number of species and substances studied.

Table S2 contains data for the distribution of pyranocoumarins; however, attention should be paid to the years of the studies. For some of them, no reliable methods for confirming the structure were available; and in other cases, minor compounds were not determined. Modern research largely focuses on determining minor compounds and studying their distribution in organs, but they do not cover all pyranocoumarin-containing plants and give no grounds to reveal the role in the body as a whole. The available data shows that folk medicine uses seeds and roots of pyranocoumarin-containing plants more often, so it can be vaguely suggested that these organs contain more such compounds, which is confirmed by some data in literature. Usually, plants accumulate substances with a protective function in seeds and roots.

Functions of pyranocoumarins in the plant are poorly studied. However, quite accurate data have been found concerning the effects of coumarins on gravitropism of roots [19], distribution of auxin [20], and protection of organs [5]. Based on papers studying their biological activity, we may suggest that pyranocoumarins play a protective role against phytopathogens [21,22]. However, this conclusion is poorly supported, since there are very few papers intended to investigate the functions of pyranocoumarins in plants.

## 5. Biological Activity of Pyranocoumarins

This paper is intended to analyze data for biological activity of plant extracts containing pyranocoumarins and individual substances from 1994 to 2022. The collected materials give a general overview of study areas and principles of influence of these unique substances on animal and human cell cultures, fungi, bacteria in vitro and on objects in vivo. For convenience, the results are distributed by various types of activity as a table and are not exhaustive (see Table 1).

### 5.1. Anti-Inflammatory Activity

Inflammation is part of a complicated biological reaction of the body to harmful irritants such as pathogens, toxins, or cell and tissue damage, and it represents a protective mechanism involving immune cells, blood vessels, and molecular mediators [62]. Without an inflammatory reaction that activates the immune system to combat pathogens, people would not be able to survive even weak infections. Therefore, inflammation fulfills a protective function in the body, but only when it is acute. Chronic inflammation ceases to be a physiological factor and becomes a pathogen causing various diseases such as hay fever, periodontium diseases, atherosclerosis, and osteoarthritis. Since inflammation is the cause and/or effect of many diseases, so searching for anti-inflammatory agents is always relevant.

Literature provides a number of papers showing anti-inflammatory mechanisms of pyranocoumarins. Son et al., from Chungnam University (Daejeon, Korea) and Kyungsung University (Busan, Korea) summarized the works and describe how decursin extracted from *Angelica gigas* suppressed the inflammatory process in mice RAW264.7 cells. The action mechanism was associated with inhibition of NF-κB transcription factor, which led to reduced expression of proteins of matrix metalloproteinase 9, monocyte chemoattractant protein 1, interleukin 8, tumor necrosis factor α, and IL-1β. Decursin increases the differentiation of IκB, a substance in the body that inhibits the activity of NF-κB, and suppresses the inflammatory response by inhibiting the differentiation and migration of NF-κB to the nucleus [23].

Praeruptorin A extracted from *Peucedanum praeruptorum* was tested for anti-inflammatory activity against RAW264.7 cells stimulated by lipopolysaccharides. Praeruptorin A in cells inhibited the production of nitrogen oxide (NO), interleukine-1β (IL-1β), and tumor necrosis factor α (TNF-α) and suppressed the expression of mRNA and proteins of inducible nitrogen oxide synthase (iNOS), IL-1β and TNF-α. Praeruptorin A reduced cytoplasmic loss of inhibitor protein κB-α (IκB-α) and thereby inhibited translocation of NF-κB from cytoplasm to the nucleus, which led to an anti-inflammatory effect [24,25]. The same effect against the signaling pathway of NF-kB translocation in RAW264.7 cell line followed by further suppression of various inflammation factors was shown by corymbocoumarin extracted from *Seseli gummiferum* subsp. *corymbosum* [26]. Reduced production of NO was also observed when administering pyranocoumarin extracted from *Clausena emarginata*, 10-(3,7-Dimethylocta-1,6-dien-3-yl)-5-methoxy-8,8-dimethylpyranocoumarin (the half maximal inhibitory concentration (IC50) = 8 µg/mL) to the mouse microglia line BV2 [27]. The reasons of such effects have not been described by the authors, but the mechanism is likely to be similar with the one for RAW264.7 cells.

The anti-inflammatory action of praeruptorin A and visnadine extracted from *Ligusticum lucidum*. Cuneifolium at 115.2 µg/cm^2^ of the area of inflammation of the skin surface and 116.52 µg/cm^2^, respectively, reduced edema in laboratory mice. Aural dermatitis in mice was induced by croton oil. Praeruptorin A decreased edema by 22% and visnadine by 43%. Visnadine differed from Praeruptorin A in the content of 3′-β-*O*-dihydroangelolic and 4′-β-*O*-acetyl residues in the pyran ring, and the authors suggest that the structure determined the increase in the anti-inflammatory effect [28].

Some artificially synthesized angular pyranocoumarins (structures of **2a**, **2b**, **3a**, **5a** are shown in the Figure S1 in Supplementary Materials) at 250 µg/mL inhibited proteinase activity. The action of pyranocoumarins was 30% to 79.72% inhibition of protein denaturation. The highest anti-proteinase activity was shown by 4 pyranocoumarins, and their action was stronger than in control aspirin (45.83 ± 4.21%). Two individual pyranocoumarins (**2b** and **3a**) with the highest activities (79.72 ± 4.51% and 74.68 ± 3.01%) were also tested in vivo on laboratory rats with formaldehyde-induced paw edema. Pyranocoumarins and control indomethacin were administered orally; as a result, paw edema decreased in rats by 29.2% (when **3a** was administered) and 6.57% (when **2b** was administered). The results of pyranocoumarin introduction gave a significantly lower effect compared with the control indomethacin (up to 72%), but this may be due to the fact that the administered dose of the active ingredient was one tenth of the indomethacin concentration given to rats. The action of pyranocoumarins towards cyclooxygenases (COX-1 and COX-2) was also tested. Cyclooxygenase is an enzyme that is responsible for formation of prostanoids, including thromboxane and prostaglandins. Inhibition of COX can provide relief from the symptoms of inflammation and pain. Most pyranocoumarin derivatives had a better effect against COX-2 (IC50 ≤ 10 µM). Compound **5a** showed significant selectivity against COX-2, which was the highest among the tested compounds (SI = 152) compared with standard celecoxib (SI = 243.9). A slightly lower activity was shown by compounds **2a** and **2b** with SI 64 and 47.3, respectively. The authors suggested that the anti-inflammatory activity of artificially synthesized pyranocoumarins is related to the amino groups in their structure [29].

### 5.2. Antioxidant Activity

Occurrence of degenerative processes is correlated with an excess of free radicals promoting oxidative processes that are harmful for the body. Free radicals have a high reactivity so they quickly deprive other molecules of electrons trying to restore their stability. Substances in the cells deprived of electrons become free radicals and also need the electron balance to be restored. As a result, a chain reaction in the body disrupts biochemical processes and destroys cells and tissues. Highly antioxidant substances neutralize free radicals. High content of compounds with antioxidant properties capable of scavenge free radicals (carotenoids, phenol, flavonoid and anthocyanin derivatives, unsaturated fatty acids, vitamins, enzymes, and cofactors) in plants has stimulated research into their use in preventive and curative herbal medicine [30].

Grandivittin, agasyllin, and aegelinol extracted from the roots of *Ferulago campestris* were tested on the cell line of polymorphonuclear neutrophils (PMN), generating active forms of oxygen in white blood cells of human whole blood (WB). Antioxidant activity of pyranocoumarins within the range of concentrations of 0.01 to 100 µg/mL was assessed on the basis of inhibition of chemical luminescence (CL). Better values were obtained for aegelinol having a hydroxyl residue at 3′ position of the pyran ring, followed by agasyllin with angeloyl at the third position of the coumarin ring and grandivittin with residual senesoyl. Inhibiting activity had linear dose-dependence showing faster inhibition in stimulation. The authors suggested that pyranocoumarins absorb the active form of oxygen or intervene with cell activation mechanisms [30].

Superoxide anion, hydrogen peroxide and radical hydroxyl are the most dangerous radicals known. The cell possesses CAT, GPx, and SOD—enzymes that remove ROS—therefore, the increase in these enzymes provides protection against the harmful effects evoked by ROS. Artificially synthesized 3′,4′-di-O-acetyl-cis-khellactone (DOAcK) was tested on laboratory rats with diabetes induced by streptozotocin. Administering DOAcK to rats increased the activity of antioxidant enzymes CAT, GPx and SOD [29]. Similar works on natural pyranocoumarins were not found.

### 5.3. Antimicrobial Activity

The question of treating bacterial and fungi infections has always been critical in the world. Wide application of antibiotic and antimycotic agents in agricultural and medical practice gave rise to the issue of antibiotic resistance of pathogen microorganisms. Moreover, antibiotics often cause allergic reactions. Finding efficient, safe, high-quality and environmentally clean plant raw materials with antibacterial activity is a topical issue.

Lee, et al., have tested the antibacterial activity of decursin and decursinol angelate extracted from the roots of *Angelica gigas* on pathogenic bacterial strains—*Escherichia coli*, *Proteus vulgaris*, *Salmonella typhimurium*, *Bacillus subtilis*, *Staphylococcus epidermis*, and *S. aureus*. The better effect was observed towards the *B. subtilis* strain for the concentration of active ingredients of 50 and 12.5 µg/mL for decursinol angelate and decursin, respectively [32].

Agasyllin and aegelinol extracted from *Ferulago campestris* were tested for various species of bacteria, showing significant activity against gram-negative and gram-positive bacteria at 16 to 125 µg/mL. The tests showed that the substances suppressed growth in ATCC strains *S. aureus*, *Salmonella thypii*, *Enterobacter cloacae*, and *E. earogenes*. Minimum inhibitory concentrations (MIC) were 16 and 32 µg/mL for aegelinol and agasyllin, respectively. Antibacterial activity of both coumarins was higher against gram-negative bacteria [33].

Pd-D-V and disenecioyl hellactone extracted from *P. decursivum* were tested against *S. sclerotiorum*, *B. cinerea*, *F. graminearum*, and *C. capsici*. Pd-DV at 30 µg/mL in the culture medium inhibited the growth of *S. sclerotiorum* by 86.4 ± 0.7 (Half maximal effective concentration (EC50) = 13.2 µg/mL) and of *B. cinerea*, *F. graminearum*, and *C. capsici* by almost 50%. Pd-D-V showed not only a wide range of effects against various bacteria, but also the best antimicrobial effect (EC50 > 56 µg/mL). Disenecioyl hellactone showed the lowest activity at the same concentration and inhibited the growth of *S. sclerotiorum* and *B. cinerea* by almost 50% (EC50 = 11.0 µg/mL), and of *F. framinearum*, *T. cucumeris* and *C. capsica* by almost 30% [34].

Artificially synthesized 3,4-angular pyranocoumarins (structures of pyranocoumarins 5a and 5c are shown in the Figure S1 in Supplementary Materials) were tested on ATCC bacterial strains: gram-positive—*S. aureus*, *B. subtilis*, *B. megaterium*, gram-negative—*E. coli* and *Pseudomonas aeruginosa*; on strains of yeast fungi from the standard collection of cultures NRRL—*S. cerevisiae* and *Candida albicans*. Screening was done by the agar diffusion test. The compounds showed high activity against gram-positive bacteria, but only two compounds (**5a** and **5c**) containing non-substituted benzal and 3,4,5-trimethoxybenzal, respectively, showed a strong effect against gram-negative bacteria (zones of inhibition (IZ): 24–26 mm). Of all the compounds, only one (**5a**) had a prominent antimicrobial activity against most tested microorganisms (IZ 22–27 mm) with a MIC of 250–500 µg/mL [29].

As antibacterial agents, pyranocoumarins can be used both in medicine and agriculture. Below are several successful works against phytopathogens.

Decursin and decursinol angelate were also tested in vitro and in vivo against pathogen fungi—*Botrytis cinerea*, *Colletotricum coccodes*, *Fusarium oxysporum f.* sp. *raphani*, *Magnaporthe oryzae*, *Rhizoctonia solani*, and *Sclerotinia sclerotiorum*, as well as two Oomycetes: *Phytophthora capsici* and *P. infestans*. The methanol extract of *A. gigas* root efficiently suppressed the development of pyricularyosis caused by *M. oryzae*, fungus from *Askomycetes*, by 75% at 100 µg/mL. Despite the low effect on the growth of fungi mycelium, the separately extracted decursin and decursinol angelate strongly inhibited the growth of *M. oryzae* spores, which was not found when using control fungicide, kasugamycin, for treatment. Among other tested pathogens, *B. cinerea* was also the most sensitive to the two pyranocoumarins: its mycelium growth was suppressed by 83%, which is similar to the effect of the control fungicide. This was followed by an in vivo test confirming the action of individual pyranocoumarins against pathogens in rice. For the in vivo test, whole rice plants were treated with decursinol angelate. When treated with 300 µg/mL decursinol angelate, the leaves were not damaged by blast. Treatment with each coumarin separately and with a combination thereof suppressed the disease by more than 80% at 100 and 30 µg/mL. A synergetic effect between the two pyranocoumarins against the pathogen was not observed. No phytotoxic consequences of adding pyranocoumarins were found, which indicates that the studied concentrations of these compounds can be safely used against fungal diseases of plants [22].

Extract of *Psoralea corylifolia* seeds with identified phenyl derivative of pyranocoumarin (PDP) was tested in vitro on *F. oxysporum*, *F. graminearum*, and *F. moniliforme*. The extract showed an inhibitory effect on the growth of individual pathogens at 10 and 1 mg/mL. Molecular modeling using trichothecene-3-O-acetyltransferase indicated affinity of PDP and the target protein, as well as the ability of pyranocoumarin to bind to the protein, thereby inhibiting the protein acetylation mechanism leading to the death of *Fusarium* sp. The authors noted that this suggestion still has to be confirmed by a more accurate analysis [21].

### 5.4. Anti-Cancer Activity

According to GLOBOCAN, 19.3 million new cancer cases and almost 9.96 million cancer deaths were recorded in the world in 2020. The global burden of cancer is expected to be 30.2 million cases in 2024, which is 56% higher than in 2020 (https://gco.iarc.fr/tomorrow/en/dataviz/isotype accessed on 15 October 2022). Figures show a high rise in emerging economies (64% to 95%) compared with developing economies (32% to 56%) due to demographic changes, though this may be aggravated by the increasing risk factors related to globalization and economic growth [63]. Cancer remains one of the most important problems of humanity, which is far from being solved. This disease has a different etiology and is characterized by different manifestations, so it is necessary to search for new methods of treatment. Secondary metabolites of plants can become prototypes of new cures for cancer. Rare compounds synthesized by plants can have useful pharmacological properties. There are a lot of potential anti-cancer agents among pyranocoumarins.

Some of the well-studied pyranocoumarins are decursin and decursinol angelate. Their anti-tumor activity was first shown in vivo in laboratory mice inoculated with tumor cells of sarcoma 180. For this purpose, decursin and decursinol angelate were sequentially injected intraperitoneally for 9 days at 50 and 100 mg/kg. Pyranocoumarin therapy of mice increased the longevity and reduced weight and volume of tumors in mice [35]. The extract of *A. gigas* containing decursin and decursinol angelate exhibited anti-cancer effect on HeLa cells through the significant activation of caspases and the cleavage of PARP. Expression of TRAIL and TRAIL receptors was also increased by two active components of *A. gigas*, respectively. In particular, two components regulated the expression of apoptosis-related proteins such as Bcl-2, Bcl-x, survivin, cIAP-1, -2, XIAP, etc. These results suggest that TRAIL expression induced by extract of *A. gigas* stimulates the extrinsic and intrinsic apoptosis pathway by activating caspase-8 and caspase-9, respectively [42]. Another test on a line of androgen-depending LNCaP showed that decursin in the medium stops G1 phase of cancer cells. This happened due to the inhibition of androgen-stimulating nuclear translocation of androgen receptors and decreased amount of androgen receptor protein [36]. An anti-tumor effect was observed in the mammary gland cancer line upon introducing decursin and decursinol angelate. Both pyranocoumarins increased the population of MCF-7 cells in G1 phase and decreased the number of cells in S phase (G1 stop) within 3.28–16.42 µg/mL. Apoptotic DNA fragmentation was found using ELISA in 24 h and using Western blot analysis of split PARP 48 h after treatment. The test showed an increased caspase-mediated apoptosis at 16.42 µg/mL decursin or 16.42 µg/mL decursinol angelate in MCF-7 [38]. Another paper attributed the anti-cancer effect to suppression of the Wnt/β-catenin pathway, counteracting the transcription of β-catenin response. Decursin also suppressed the expression of D1 cyclin and c-myc, which are β-catenin target genes, so that pyranocoumarin inhibited the growth of PC-3 prostate cancer cells [39]. For RC-58T/h/SA#4 prostate cancer cells, decursin reduced the cell proliferation on a dose-dependent basis (6.57; 13.14; 19.70; 26.27 and 32.84 µg/mL) for 48 h. Cancer cells were more sensitive to decursin than normal cells. Stain testing of Hoechst 33,258 cells indicated that decursin promoted condensation of chromatin and apoptotic bodies [40]. Decursin also exhibited a cytotoxic effect in combination with bortezomib in human multiple myeloma cells. Decursin inhibited the viability of U266, MM.1S, and ARH77 cells, but not of peripheral blood mononuclear cells (PBMC). Decursin-induced apoptosis was mediated by activation of caspase-8, -9, and -3 and by inhibition of constitutive STAT3 activation by inhibiting activation of Janus-activated kinase 2 (JAK2) in U266 cells. Moreover, decursin inhibited interleukin-6-induced STAT-3 activation on a dose-dependent basis in MM.1S cells. Due to these properties, decursin significantly amplified the apoptotic properties of bortezomib against U266 cells [41]. *A. gigas* extract containing decursin and decursinol angelate showed an inhibitory action on HeLa cervical cancer cells with up to 50% cancer cell proliferation at 6.57 µg/mL. An anti-proliferative action of decursin and decursinol angelate on normal cells of the A549 or AGS lines was not observed at the same concentration. This mechanism was attributed to the fact that the extract increases the expression of TRAIL that stimulates extrinsic and intrinsic apoptosis pathways by activating caspase-8 and caspase-9 [42].

Decursinol extracted from *Saposhnikovia divaricata* suppressed the growth of DU-154 prostate cancer cells (GI50 3.09 µg/mL), was more efficient compared with the standard drug cisplatin, and showed high activity towards MEL-8 melanoma cells (GI50 = 3.46 µg/mL) [43].

Clausarin extracted from *Clausena excavate*, as well as a number of synthesized pyranocoumarins, were tested for the cytotoxicity against A549, MCF-7, KB, and KB-VIN human tumor cells. Clausarin showed the best anti-tumor effect against A549, KB, and KB-VIN with EC50 from 1.59 to 2.98 µg/mL. Clausenidin and two other synthesized compounds demonstrated a significant level of activity only towards KB-VIN cells with EC50 from 2.25 to 2.87 µg/mL, while the activity was insignificant or none at all against other lines. The study results are interesting since KB-VIN cells has multiple drug resistance [44].

(±)-Praeruptorin A and (±)-praeruptorin B extracted from *Peucedanum praeruptorum* were tested for SGC7901 human stomach cancer cells. The study showed that (±)-praeruptorin A increased sensitivity to drugs allegedly by inhibiting the expression of *P*-glycoprotein responsible for drug efflux from cancer cells via ATF-depending pumps. Moreover, the cell growth was decreasing faster in combined treatment with pyranocoumarin and doxorubicin than when using the chemotherapeutic agent alone, which suggests that (±)-praeruptorin A can reduce the doxorubicin dose to achieve the desired effects [45].

To confirm the activity of pyranocoumarins extracted from *P. praeruptorum* roots, the permeability of cells by calcein-AM was considered relative to MES-SA/Dx5 poly-drug resistant cancer cells. Five angular pyranocoumarins at 10 µM each showed a significantly higher activity compared with known inhibitors (verapamil and ciclosporin A) [25].

Grandivitin extracted from *Ferulago macropara*, inhibited matrix metalloproteinase 9 (MMP9). Matrix metalloproteinases, a large family of extracellular zinc-dependent endopeptidases, play a major role in the ischemic cell injury. Their targets include other proteinases, proteinase inhibitors, clotting factors, chemotactic molecules, latent growth factors, growth factor-binding proteins, cell surface receptors, cell–cell adhesion molecules, and virtually all structural extra-cellular matrix proteins. Grandivitin suppressed protein fluorescence binding to it in the hydrophobic cavity and thereby changed the secondary and tertiary MMP9 structures. The biological significance of this work is evident because MMP9 serves as a potential target protein for anticancer agents. The binding study of grandivitin with MMP9 is of great importance in pharmacy, pharmacology and biochemistry [45].

Clausenidin was also tested on HepG2 hepatocellular carcinoma cells. After being introduced into the medium, clausenidin increased the caspase-8 activity and expression of protein components in the death-inducing signaling complex (DISC) in HepG2 cells. The ultra-structural analysis found morphological abnormalities typical for apoptosis. Moreover, clausenidin reliably reduced expression of the vascular endothelial growth factor (VEGF) [47]. Another group of researchers have tested the cytotoxicity of clausenidin extracted from *C. excavata* on the colon adenocarcinoma line HT29 and normal colon cell line CCD-18Co. Clausenidin in DMSO and in combination with hydroxypropyl-β-cyclodextrin (Clu/HPβCD) was used for the study [48]. Cyclodextrins can increase the water-solubility of low-soluble substances and improve drug penetration through biological membranes [64]. The cytotoxic action of clausenidin in combination with HPβCD against cancer cells was stronger than when dissolved in DMSO. When comparing the action with normal cells, the side effect was reduced. The authors noted that the Clu/HPβCD complex initiated cytotoxicity mediated by active oxygen forms in HT29 cells, which resulted in a stopped cell cycle and death caused by apoptosis associated with caspase activation [47].

Anti-cancer activity was primarily shown for linear pyranocoumarins. It should be noted that the search found no papers studying the action mechanism of angular pyranocoumarins. The two forms of angular pyranocoumarins and the kinetics of linear pyranocoumarins are still to be studied. Dimeric pyranocoumarins—calanolides and condensed pyranocoumarins—also show great potential for future use.

### 5.5. Antivirus Activity

Antiviral activity is not as widely studied for pyranocoumarins as bacterial infections or cancer. However, the latest global events have become an incentive to search for compounds with antiviral activity.

Causenidin and nordentatin extracted from *C. excavata* were tested on HepG2 cells that were infected with hepatitis B. Among the tested pyranocoumarin analogs, causenidin and nordentatin had anti-HBV values EC50 of 0.62 µg/mL and 2 µg/mL, respectively. Moreover, other pyranocoumarins were chemically synthesized, among which four compounds showed antiviral activity [44].

A number of artificially synthesized pyranocoumarins were tested on the cell line of a kidney of the African green monkey (CV-1) infected with measles. Some were rather specific and strong MV inhibitors with EC50 from 0.2 to 50 µg/mL, while most EC50 value were below 5 µg/mL. The compounds inhibited nine strains of the measles virus, while the drugs did not physically destroy virion in virulence tests in order to inhibit virus replication [49].

### 5.6. Antihyperglycemic and Antidyslipidemic Activity

Obesity and overweight have increased at an alarming rate in the world during the last three decades. Obesity is a crucial factor in the development of metabolic abnormalities, including glucose intolerance, insulin resistance, metabolic syndrome, low-grade inflammation and oxidative stress [7]. To reduce obesity-associated risk factors, various methods for prevention and treatment are used, including pharmaceuticals. Disadvantages of conventional synthetic drugs include low efficacy, narrow therapeutic range, toxicity, and major side effects. Plant-derived drugs are safer, and many papers show that using plants against obesity is highly effective [65].

Taira, et al., showed that pyranocoumarins extracted from *Peucedanum japonicum* Thunb. inhibited lipid accumulation and lipogenous expressions of genes in 3T3-L1 adipocytes [50].

Our experiments on 3T3-L1differentiated adipocytes with seven coumarins from *P. sibiricus* proved that the total content of triacylglycerols in cells treated with dihydrosamidin was lower at 10 µg/mL (22.6% of the control level) and at 20 µg/mL (19.8% of the control level). The primary coumarin of *P. sibiricus* roots is an active agent against obesity and can be used against overweight [51].

Hossin, et al., evaluated oral consumption of pure dihydropyranocoumarins (DPC) extracted from *P. japonicum* in mice fed a high-fat diet and studied their activity when nanoparticles are added. Weight gain and accumulation of fat in white fat tissues were significantly suppressed when DPC was consumed with food (1.943 mg/mouse/day). Consumption of dihydropyranocoumarins significantly decreased the average size of adipocytes and increased the mRNA levels of genes associated with thermogenesis. Nanoparticulation of DPCs with polylactic-co-glycolic acid (PLGA) dramatically increased its activity almost 100-fold over that of a non-nanoparticulated form. Wherein, the results showed activity of dihydropyranocoumarins against obesity in vivo and suggested that encapsulation of PLGA nanoparticles was useful to amplify the DPC activity against obesity in order to develop natural and safe anti-obesity agents [52].

(+)-trans-Decursidinol, Pd-C-I and Pd-C-II, Pd-C-III were extracted from whole *Angelica decursiva* plants and tested on the HepG2 cell line. The presence of these compounds in the medium increased glucose absorption and reduced expression of PTP1B in insulin-resistant HepG2 cells. (+)-trans-decursidinol showed a high inhibitory action towards PTP1B among extracted compounds with IC50 0.61 µg/mL, which exceeded the action of the control ursolic acid three times (IC50 = 3.14 µg/mL). Pd-C-I, Pd-C-II, and Pd-C-III showed inhibitory activity against PTP1B with IC50 values of 1.35, 2.03, and 4.60 µg/mL, respectively. Coumarins inhibited albumin nitration mediated by peroxynitrites (ONOO-) [66].

### 5.7. Other Activities

Other biologically active properties of pyranocoumarins, which cannot be classified as any of the activities given above, are considered below. Visnadine extracted from *Ammi visnaga* fruits was tested on guinea pigs with muscular contractions induced by norepinephrine (16.92 µg/mL). Visnadine predominantly suppressed retractive reactions of K^+^-spasms in aorta segments mediated by Ca^2+^ penetration via Ca^2+^ [53].

The group led by Gulyayev and Urbanova has studied neuropharmacological effects of *P. sibiricus* extracts. An aqueous infusion of *P. sibiricus* showed anxiolytic properties, supposedly, by activating GABA_A_ receptor. The GABA_A_ receptor is an ionotropic receptor and ligand-gated ion channel. Its endogenous ligand is γ-aminobutyric acid (GABA), the major inhibitory neurotransmitter in the central nervous system [54]. It has been found that course administration of a *P. sibiricus* infusion at 0.05 mL/kg limits the development of emotional and neurological disturbances in case of cerebral ischemia in rats [55]. Moreover, *P. sibiricus* extract was found to inhibit hemolysis of red blood cells induced by peroxide solution, thereby showing the membrane-stabilizing action of coumarins. The best inhibitory effect was shown at 1 µg/mL [56]. The extract was also injected into laboratory rats at 100–400 mg/kg, which had an anti-anxiety action, increased the length of thiopental-induced sleep, and had an anticonvulsant action. This indicates the neuromodulatory activity of the extract, supposedly caused by activating the GABA system by coumarin compounds [57].

(±)-Praeruptorin extracted from *P. praeruptorum* was tested on laboratory mice with drug-induced asthma. Injection of (±)-praeruptorin suppressed inflammation of airways, hyperactivity and remodeling of airways, reduced IL-4 and IL-13 in BALF and IgE in serum, inhibited TGF-β1 and pSmad2/3 expression, increased Smad7 expression in lung tissue, and increased INF-γ levels in BALF. Due to these processes, mice showed improved condition of airways resulted from their reduced inflammation [58].

Out of seven pyranocoumarins extracted from *Clausena emarginata*, compounds of clauemarmarin C, D and two known analogs at 10 µM showed hepatoprotective activity against DL-galactosamine-induced damage to the diploid epithelial cell line of normal liver in adult rats with phenotype properties of oval cells WB-F344 [59].

Mammeasins E and F extracted from *Mammea siamensi* were tested relative to 5α-reductase enzyme. Out of all tested pyranocoumarins, mammeasins E and F caused the highest inhibition of 5α-reductase of testosterone [67].

cis-Khellactone, d-laserpitin, isolaserpitin, and octanoyllomatin extracted from *Seseli devenyense* were administered to zebrafish larvae. There was an anxiolytic effect of pyranocoumarins defined by the decreasing residence time within the living space of larva (reverse thigmotaxis) [60].

From n-hexane extract of the above-ground parts of *Seseli petraeum*, pyranocoumarin was extracted, which was called 3′-isovaleryl-4′-oxolomatin (or petracoumarin), as well as 12 previously known pyranocoumarins such as: octanoyllomatin, selinidin, anomalin, 3′-isobutyllomatin, 3′-angeloyl-4′-isovaleryl-cis-hellactone, 3′-isovaleryl-4′-angeloyl-cis-hellactone, calipterixin, samidin, 4′-senecioyl-cis-hellactone, 3′-senecioyl-cis-hellactone, cis-hellactone, and angelicin. Several coumarins were tested for their inhibitory activity against α-amylase and α-glycosidase, and were found to show prominent inhibitory activity against α-glycosidase and a low inhibitory potential against α-amylase. Octanoyllomatin was the best inhibitor of a-glycosidase (IC50 = 69.00 ± 0.43 µg/mL) [61].

Thus, pyranocoumarins are promising antioxidants, anticancer agents, and also show antibacterial, antiglycemic, and neuroprotective effects. However, despite a large number of studies on the various biological activities of various pyranocoumarins, there is practically no data on their bioavailability. It is expected that this issue will be the task of subsequent preclinical trials.

## 6. Prospects for the Pyranocoumarins Study

The study of pyranocoumarin formation in the cultural medium of plants shows great potential. Using cell cultures in vitro helps study the formation of specialized exchange compounds in controlled conditions and define regulatory mechanisms and systems of this process. The fact that there is no deficiency of materials is equally important. There are certain examples of pyranocoumarin identification in a cell culture; however, regulatory mechanisms were not studied.

We obtained callus and suspension cell cultures from aseptic seedlings of *Phlojodicarpus sibiricus*. The callus cell cultures of *P. sibiricus* were characterized by white-yellow color, combination of loose and dense aggregations of cells, and satisfactory growth: growth index by dry biomass for a culture of leaf origin was 7–9, hypocotyl origin 10–12, and root origin 11–13. Suspension cell cultures were initiated from callus cultures of leaf and hypocotyl origin, which also had white-yellow color and predominantly consisted of cell aggregates of meristem-like and parenchyma-like types, while the aggregation degree differed for cultures of different origin. Cell viability during the growing cycle was 70–80%. Unlike the initial callus cultures, the highest growth (growth index of about 10) was shown by the line of suspension cell culture obtained from calluses of leaf origin. Preliminary phytochemical screening using UPLC-MS showed the presence of coumarins of the kellactone group in the biomass of primary (1st–3rd growth cycle) callus and suspension cell cultures of *P. sibiricus* [68]. This was followed by studies of suspension cultures of *P. sibiricus* in long-term growing In vitro. The highest growth characteristics were shown by the suspension culture of leaf origin: growth indexes by various categories (dry and wet cell biomass, cell concentration) I = 10–14; specific growth rate μ = 0.3–0.4 day^−1^; maximum dry biomass accumulation M = 9.6 g/L; economic coefficient Y = 0.29. The cell culture of hypocotyl origin had lower growth characteristics: I = 3.6–4.9, μ = 0.12–0.18 day-1, M = 6.6 g/L, Y = 0.16. Differences in the growth of the studied cultures correlate with the aggregation degree of cells: the suspension culture of cells of leaf origin consists mainly of fine aggregates (10 to 30 cells) while the culture of hypocotyl origin is represented by coarse aggregates (no less than 50 cells in the aggregate). A fine suspension culture of *P. sibiricus* cells of leaf origin was grown in two types of laboratory bioreactors—a bubble column and a stirred tank. It was found that growing in the bubble column bioreactor is associated with improved primary growth characteristics (growth index by dry biomass I = 12.7; productivity by dry biomass P = 0.78 g/L × day, μ = 0.18 day^−1^, M = 15.8 g/L, Y = 0.49). When using the stirred tank bioreactor, there was a noticeable reduction in growth characteristics, which is evidently due to the damaging action on the cells by agitation devices [69]. Phytochemical analysis showed significant differences in the composition of phenolic compounds in cells in vitro and in roots of the plant. The cultures of *P. sibiricus* cells predominantly contained polar (hydrophylic) compounds classified as phenol derivatives—coumarin and benzofuran glycosides. The main components in the roots were more hydrophobic metabolites—ethers of pyranocoumarin kellactone. The analysis of the phytochemical composition of suspension cell cultures of *P. sibiricus* showed significant differences in the composition of phenol compounds in cells in vitro and in roots of the intact plant. Suspensions cell cultures of *P. sibiricus* contained predominantly polar (hydrophylic) compounds wherein the main components in the roots were hydrophobic metabolites. Most compounds detected in cells in vitro (except for glycoside kellactone [70]) are not characteristic of intact plants of *P. sibiricus* [51].

Some of the detected compounds (hexoside ether of prenylated coumarin osthenol/7-dimethylsuberosin, malonyl derivatives of coumarin glycosides) can be classified as quite rare phenol derivatives of plants. Other detected compounds (benzofuran glycosides) are widely spread among various taxons [70,71,72,73,74,75]; however, the available literature apparently contains no information about their presence in *Phlojodicarpus* spp. [69]. Curiously, prenylated coumarins characteristic of the intact plant (visnadine, dihydrosamidine, and other kellactone ethers) were identified in the initial callus cultures of *P. sibiricus*, but were not found in long-grown suspension cell cultures [68,69]. The loss of pyranocoumarin synthesis by cell cultures is most likely associated with a change in the strategy for the existence of plant cells in vitro. Heterotrophic nutrition and the absence of the need to protect against pathogens lead to a change or reduction in substances of specialized synthesis. However, further experiments in the form of changing the nutrient medium, the use of elicitors, and transformation can give positive results.

These results are well aligned with the available data concerning changes (in comparison with intact plants) of secondary metabolism in plant cells cultivated in vitro. Such trends were noted in the formation of steroid glucosides in cell cultures of *Dioscorea deltoidea* Wall. and *Tribulus terrestris* L. Both cultures only showed accumulation of furostanol glycosides promoting cell proliferation, whereas spirostanol forms of steroid glycosides are more characteristic of intact plants [76,77].

The literature shows at least 10 cell cultures and plant organs being pyranocoumarin producers (see Table 2). However, with the exception of our papers and Xu, et al., no pyranocoumarins were found in cell cultures [68,69,78]. For example, the culture of *A. visnaga* cells was independently obtained by three groups of researchers, but no secondary metabolites except for synthesis of furanochromones were found [79,80,81,82].

Using cell cultures and tissues as a method for obtaining renewable high-quality plant raw materials for producing biologically active compounds from plants, namely, for creating new and safe functional drugs, has great potential, as well as a number of advantages, for example, a radical solution to the deficiency problem. The produced raw materials are standard, irrespective of weather and climatic conditions, and completely free of all types of contaminations, since the biomass of cell and tissue cultures is grown in sterile and controlled conditions of a laboratory and/or factory [101,102].

In recent years, great success was achieved in metabolic engineering of microorganisms for the purpose of producing valuable compounds of plant origin [103,104]. Levopimaradien and other diterpenoids were successfully obtained in *Escherichia coli* cells [105]; artemisinic acid in *Saccharomyces cerevisiae* yeasts [106]; dihydroquerticine in *E. coli* [107], cannabinoids in *S. cerevisiae* [108], taxadiene in *E. coli* [109], noscapine in *S. cerevisiae* [110] and opioids in *S. cerevisiae* [111]. Studies of the biosynthesis pathways of secondary metabolites in cell cultures have both fundamental and applied significance irrespective of the source of raw materials. First of all, targeted regulation of biosynthesis becomes possible. Second of all, the known enzyme genes of biosynthesis pathways can be transferred to other organisms (bacteria, fungi, and other plants and/or cells thereof). The production of secondary metabolites in microorganisms is an environmentally clean and renewable method for obtaining the target product compared with chemical synthesis. Therefore, creation of producing microbes for the rapid production of secondary synthesis compounds using metabolic engineering and synthetic biology is rather relevant. A compelling argument for using the bacterial system is the phenylpropanoid pathway in the bacterial cell, as well as the significant reinforcement thereof by adding the signal peptide of cytochrome P450 (TT7) from *Arabidopsis thaliana* [107]. Successful transformation of primary genes of prenyltransferases cytochrome P450 participating in the biosynthesis of pyranocoumarins into cells of *S. cerevisiae* and *E. coli* is shown in the paper by Bu, et al., This paper helped identify the enzymes responsible for the formation of the pyran ring [112].

## 7. Conclusions

The ability to synthesize various substances of specialized metabolism is a unique characteristic of plants that has allowed them to survive and occupy vast areas. Various groups of secondary metabolites provide protection, attract pollinators, and participate in the regulation of life processes. The variety of substances synthesized by plants helped people produce incenses, dyes, spices, and multiple other essential products that have become common today. Medicines are the most important and relevant ones among them. Searching for unique medicinal drugs in flora remains one of the central tasks of the pharmaceutical industry.

Simple coumarins are widely distributed and fulfill a general protective function. Unlike them, pyranocoumarins belong to a rare sub-group of secondary metabolites synthesized in two families only. This narrow evolutionary adaptation is not random, but most probably caused by the need for protection against a certain pathogen. Selective accumulation of pyranocoumarins in seeds and roots indicates their protective function. This hypothesis has been proven by Yoon and Srinivasan, et al. [21,22]. However, this area requires a deeper study due to the lack of data on pyranocoumarin-containing plant species.

It is rather interesting that the biosynthesis pathway of pyranocoumarins, as any other phenol compounds, starts with the phenylpropanoid pathway, and a proven precursor of all pyranocoumarins is umbelliferon, a coumarin widely spread in the flora. The formation of linear pyranocoumarins from 7-dimethyolsuberoside and 7,8-angular pyranocoumarins from osthenol is well-studied. However, not much is known about the other two, quite rare, 4,5- and 5,6-pyranocoumarins, which are the subject matter of future research.

Pyranocoumarins have various biological activities, but the most interesting and relevant today is anticancer activity. Numerous studies have reliably proven the activity of linear coumarins against various cancer lines. For angular pyranocoumarins, there are fewer experimental works on the action mechanisms, but there are data showing that the growth of cancer cells can be slowed down without significant adverse effects on normal cells. These results are a good prerequisite for the identification of action mechanisms and the development of new drugs. Thus, the information presented in this review indicates the demand for natural pyranocoumarins as promising compounds with a variety of effects on the human body deserving further close study. Unfortunately, many plants that have shown high production of pyranocoumarins (*Phlojodicarpus sibiricus*) or rare pyranocoumarins (as a group of mammeosines) are endangered or endemic species, which complicates pharmaceutical production. Cell cultures can be a good alternative. If substances play a protective role, they are most likely toxic, and it is rather difficult to achieve their high production in cell culture, where the principle of existence is designed only for cell division. To date, the number of studies on the synthesis of pyranocoumarins in cell cultures is insufficient, but is gradually increasing. Accordingly, it is necessary to carry out targeted studies on the induction/cultivation of organ tissue cultures and whole plants producing pyranocoumarins

## Figures and Tables

**Table 1:** Biological activities of pyranocoumarins.

Activity	Pure Substance/Extract	Pyranocoumarins Form	Model	Mechanism of Action	References
The anti-inflammatory activity	Decursin from *Angelica gigas*	L *	In vitro in macrophages	Decursin suppressed the expression of proteins matrix metalloproteinase 9, monocytic chemoattractant protein 1, interleukin 8, tumor necrosis factor α and IL-1β due to the fact that it inhibited the activity of the NF-kB translocation signaling pathway.	[23]
	Praeruptorin A from *Peucedanum praeruptorum*	Ad *	In vitro in murine macrophage of RAW264.7 cell line	Pyranocoumarin suppressed the cytoplasmic loss of inhibitor κB-α protein and inhibited the translocation of NF-κB from cytoplasm to nucleus.	[24]
	(+)-Praeruptorin A from *P. praeruptorum* roots	Ad	In vitro in murine macrophage of RAW264.7 cell line	The presence of (+)-praeruptorin A in medium reduced nitric oxide production in cells.	[25]
	Corymbocoumarin from *Seseli gummiferum* subsp. *corymbosum*	Ad	In vitro in murine macrophage of RAW264.7 cell line	The presence of corimbocoumarin in medium suppressed the nuclear factor κB (NF-κB) signaling pathway activation and heme oxygenase (HO)-1 expression induction in cells.	[26]
	10-(3,7-Dimethylocta-1,6-dien-3-yl)-5-methoxy-8,8- dimethylpyranocoumarin, nordentain from *Clausena emarginata*	L	In vitro in murine microglial of BV_2_ cell line	The presence of pyranocoumarin in medium reduced the nitric oxide production in cells.	[27]
	(+)-Praeruptorin A, visnadin from *Ligusticum lucidum* subsp. *cuneifolium*	Ad	In vivo in mice	(+)-Praeruptorin A reduced local edema by 22%, visnadin, by 43%. The anti-inflammatory activity of these compounds appears to be modulated by the substituents on their aromatic ring.	[28]
	Artificially synthesized 3,4-angular pyranocoumarins	A *	Against proteinase enzymes	Proteinase activity inhibition. The best effect was shown by pyranocoumarins with amino groups in the pyran ring	[29]
			In vivo in rats	Rat paw edema decreased to 29.2%.	
			Against COX-1 and COX-2	Most of the pyranocoumarin derivatives showed themselves against COX-2 (IC50 ≤ 10 µM).	
Antioxidant activity	Grandivittin, agasyllin, aegelinol from *Ferulago campestris*	L	In vitro in human polymorphonuclear neutrophils (PMN) respiratory burst cells and on human whole blood leukocytes (WB)	It is suggested that pyranocoumarins scavenge reactive oxygen species or interfere with cellular activation mechanisms.	[30]
	Artificially synthesized 3′,4′-Di-*O*-acetyl-cis-khellactone (DOAcK)	Ad	In vivo in rats	DOAcK administration increased the activities of Catalase, Glutathione Peroxidase and Super Oxide Dismutase.	[31]
Antimicrobial activity	Decursin and decursinol angelate from *Angelica gigas*	L	In vitro in *Bacillus subtilis*	The six-membered ring and senecioylic acid type side chain closely related to the enhanced antibacterial activities of coumarins against *B. subtilis*. Although a precise mechanism has not yet been clarified, these phenomena might be considered to occur due to the differences in the binding affinities of both compounds on the active sites of the enzymes or receptors from the differences in the position of the side chain moiety.	[32]
	Agasyllin, aegelinol from *Ferulago campestris*	L	In vitro in bacteria	At a concentration of 16 to 125 µg/mL, the growth of *Staphylococcus aureus, Salmonella thypii, Enterobacter cloacae* and *Enterobacter earogenes* was suppressed, from 5 to 25 µg/mL—*Helicobacter pylori.*	[33]
	Pd-D-V and Disenecioyl Khellactone from *P. decursivum*	L	In vitro against *S. sclerotiorum*, *B. cinerea*, *F. graminearum* and *C. capsici*	Pd-DV at a concentration of 30 μg/mL in medium inhibited the growth of *S. sclerotiorum* by 86.4 ± 0.7, and also inhibited the growth of *B. cinerea*, *F. graminearum* and *C. capsici* by almost 50%.	[34]
		Ad	In vitro against *S. sclerotiorum*, *T. cucumeris*, *B. cinerea* and *F. graminearum*	Disenecioyl Khellactone at a concentration of 30 μg/mL inhibited the growth of *S. sclerotiorum* and *B. cinerea* by almost 50%, and the growth of *F. framinearum*, *T. cucumeris* and *C. capsica* by almost 30%.	
	Agasyllin, aegelinol from *Ferulago campestris*	L	*Magnaporthe oryzae* in vitro and in vivo	Pyranocoumarins inhibited *M. oryzae* spore germination and upressoria at concentrations of 50 and 200 µg/mL and prevented rice disease by more than 80% at 100 and 300 µg/mL.	[22]
	Phenyl derivative of pyranocoumarin (PDP) from *Psoralea corylifolia* L.	L	In vitro in *Fusarium oxysporum*, *F. graminearum*, and *F. moniliforme*	The ligand PDP showed bifurcated hydrogen bond interaction with active site residues at TYR 413 and a single hydrogen bond interaction at ARG 402 with a docking score −7.19 and glide energy of −45.78 kcal/mol. This indicated a strong binding of the ligand with the trichothecene 3-O-acetyltransferase, preventing as a result the acetylation of the trichothecene mycotoxin and destruction of the “self-defense mechanism” of the *Fusarium* sp.	[21]
	Artificially synthesized 3,4-angular pyranocoumarins	A	In vitro in bacteria and fungi	Most pyranocoumarins exhibited inhibitory activity in zones with diameters in the range of 15–19 mm or more. It is assumed that activity is associated with lipophilicity of molecules.	[29]
Anticancer activity	Decursin and decursinol from *A. gigas*	L	In vivo in mice	Tumor size decreased by 40.6% and 45.6% with introduction of decursin and decursinol angelate	[35]
			In vitro in prostate cancer DU145 and LNCaP cells	Decursin inhibited cell growth by stopping the G1, G2 and S phases at different dosages (25–100 μM)	[36]
			In vitro in LNCaP cells	Decursin inhibits androgen-stimulated nuclear translocation of the androgen receptor and reduces the amount of androgen receptor protein.	[37]
			In vitro in MCF-7 cells	Decursin and decursinol angelate exerted growth inhibitory effects on MCF-7 cells through G1 arrest and caspase-mediated apoptosis.	[38]
			In vitro in prostate cancer PC-3 cells	Decursin suppressed cell proliferation by suppressing Wnt/β-catenin pathway.	[39]
			In vitro in RC cells	Decursin inhibits cell proliferation by inducing apoptosis, which is mediated by both caspase-dependent and caspase-independent apoptosis pathways.	[40]
			In vitro in U266, MM.1S, ARH77 cells	Decursin showed a synergistic effect with bortezomib due to inhibition of STAT3 (activation of signal transducers and the activator of transcription 3)-induced proliferative and angiogenic effect in multiple myeloma.	[41]
			In vitro in HeLa cells	Extract increases expression of TRAIL, which stimulates extrinsic and intrinsic pathways of apoptosis through the activation of caspase-8 and caspase-9, respectively.	[42]
	(+)-Decursinol from *Saposhnikovia divaricata*	L	In vitro in MEL-8, U-937, DU-145, MDA-MB-231 and BT-474 cell lines	It inhibited growth and proliferation of DU-154 prostate cancer cells and MEL-8 melanoma cells. The mechanism has not been considered.	[43]
	Clausarin from *Clausena excavata*	L	In vitro in the multi-drug resistant cell line KB-VIN cells	The mechanism(s) of action should be further investigated.	[44]
	Artificially synthesized linear pyranocoumarins	L			
	(±)-Praeruptorin A & (±)-Praeruptorin B from *Peucedanum praeruptorum*	Ad	In vitro in SGC7901 cells	(±)-Praeruptorin A increases cancer cells drug sensitivity, presumably by inhibiting the expression of P-glycoprotein, which is responsible for the efflux of drugs from cancer cells through ATP-dependent pumps.	[45]
	Angular pyranocoumarins from *Peucedanum praeruptorum* roots	Ad	In vitro in MES-SA/Dx5	Pyranocoumarins inhibited drug efflux via MDR protein, which prevents multidrug-resistant cancer formation.	[25]
	Grandivitin from *Ferulago macropara*	L	Molecular modeling analysis against MMP9	By binding to Matrix metalloproteinase 9 (MMP9), it affected secondary structure and modified tertiary structure of this protein.	[46]
	Clausenidin from *Clausena excavata*	L	In vitro in HepG2 cells	Clausenidin increased the activity of caspase-8 and expression of protein components of the death inducing signaling complex (DISC) in HepG2 cells.	[47]
		L	In vitro in HT29 and CCD-18Co	HT29 colorectal adenocarcinoma cells treated with clausenidin/hydroxypropyl-β-cyclodextrin complex showed cell cycle arrest and death by apoptosis associated with caspase activation.	[48]
Antiviral activity	Causenidin and nordentatin from *C. excavata*	L	In vitro in HepA2 cells	The pyranocoumarins suppressed hepatitis.	[44]
	Artificially synthesized linear pyranocoumarins	L		B virus surface antigen in HepA2 cells and had anti-HBV values. Their EC50 values were 1.14, 1.34, 1.64 and 1.63 µM.	
	Artificially synthesized 5,6-angular pyranocoumarins	A	African green monkey (CV-1) kidney cell lines infected with measles virus	The compounds inhibited nine strains of measles virus, and in virucidal tests, drugs did not physically destroy virion to inhibit virus replication.	[49]
Antihyperglycemic and antidyslipidemic activity	Pyranocoumarins isolated from *P. japonicum*	Ad	In vitro on 3T3-L1 adipocytes	Inhibited lipid accumulation and lipogenic gene expression in 3T3-L1 adipocytes.	[50]
	Root and herb extracts of *P. sibiricus*, separately isolated fractions of dihydrosamidine, kelactone esters	Ad	In vitro in 3T3-L1 cells	Root extracts and esters of kellacton inhibited triacylglycerol accumulation, while activity depended on acyl groups type.	[51]
	Dihydropyranocoumarins isolated from *P. japonicum*	Ad	In vivo in mice	Consumption of dihydropyranocumarins significantly reduced average size of adipocytes and increased mRNA levels of genes associated with thermogenesis. Nanoparticulation of DPCs with polylactic-co-glycolic acid (PLGA) dramatically increased its activity almost 100-fold over that of a non-nanoparticulated form.	[52]
	(+)-trans-decursidinol, Pd-C-I and Pd-C-II, Pd-C-III isolated from *Angelica decursiva*	L	In vitro in HepG2 cells	Pyranocoumarins increased glucose uptake and decreased PTP1B expression in insulin resistant HepG2 cells; inhibited albumin nitration mediated by ONOO-, removed peroxynitrite, ROS.	[30]
Other activities/effects	Visnadin from *Ammi visnaga*	Ad	In vitro in aortic segments of guinea-pigs	Visnadin predominantly inhibits contractile responses mediated by penetration of Ca^2+^ through Ca^2+^.	[53]
	Tincture from *Phlojodicarpus sibiricus*	Ad	In vivo in rats	Tincture showed anxiolytic properties, presumably due to GABAA benzodiazepine receptors activation.	[54]
	Tincture from *P. sibiricus*	Ad	In vivo in rats	The extract limits the development of emotional and neurological disorders in cerebral ischemia in rats.	[55]
	Extract of *P. sibiricus*	-	In vitro in erythrocites	Extract inhibited hemolysis of erythrocytes, which is presumably due to the membrane-stabilizing properties of coumarins.	[56]
	Extract of *P. sibiricus*	-	In vivo in rats	Extract has a neuromodulatory effect, probably due to activation of GABA (gamma aminobutyric acid)-ergic system by coumarin compounds.	[57]
	(±)-Praeruptorin from *P. praeruptorum*	Ad	In vivo in mice	(±)-Praeruptorin A suppressed airway inflammation, airway hyperreactivity and remodeling, reduced serum IL-4 and IL-13 levels in BALF and IgE, inhibited TGF-β1 and pSmad2/3 expression, increased Smad7 expression in lung tissue, and also increased INF-γ levels in BALF.	[58]
	Clauemarmarin C, D & 5-hydroxy-8,8-dimethyl-10- (7-hydroxy-3,7-dimethylocta-1,5-dien-3-yl) pyranocoumarin from *C. emarginata*	L	In vitro in WB-F344 cells	They showed a hepatoprotective effect, the mechanism was not considered.	[59]
	Mammeasins E and F from *Mammea siamensis*	C *	Against enzyme testosterone 5α-reductase	Inhibited testosterone 5α-reductase.	[21]
	cis-Khellactone, d-laserpitin, isolaserpitin and octanoyllomatin, isolated from *Seseli devenyense*	Ad	In vivo on zebrafish larvae	Coumarins reduced anxiety behavior (anxiolytic activity) in zebrafish larvae. The mechanism was not considered.	[60]
	Angular pyranocoumarins from *Seseli petraeum*	Ad	Against enzymes α-amylase and α-glucosidase	The coumarins exhibited notable inhibitory activity against the α-glucosidase enzyme and low inhibitory potential against α-amylase.	[61]

* L—linear 6,7-pyranocoumarin; Ad—angular 7,8-pyranocoumarin; A—angular 5,6- or 7,8-pyranocoumarins; C—condensed 4,5-pyranocoumarin.

**Table 2:** Cell cultures of pyranocoumarin producers.

Species	Explant	Growth Conditions, Media	Results	Pyranocoumarins	References
***Umbelliferae (Apiaceae)***					
*Ammi visnaga* (L.) Lam	Fruits	MS * with 1.0 mg/L 2,4-D *	Callus tissue contained 45 mg visnagin/1100 g dry weight.	Not detected	[79]
	Fruits	1. MS with 1.0 mg/L 2,4-D 2. MS with 10 μM L-glutamine and 1.0 mg/L 2,4-D, 0.5 mg/L BA *, or a growth regulator-free MS.	1. Globular masses. 2. The globular, heart and torpedo embryos of *A. visnaga* produced khellin and visnagin.	Not detected	[80]
	Hypocotyl of sterile seedlings	MS with 2.5 mg/L NAA * and 1.0 mg/L BA	Callus tissue with visnagin and/or khellin.	Not detected	[81]
	Seeds	MS with 1.0 mg/L BA and 2.0 mg/L 2,4-D	Microshoots in vitro and callus culture.	Not detected	[82]
*Ammi majus* L.	Not specified	Linsmaier-Skoog’s media with NAA and BA	Tissue and organ cultures containing different concentrations of the linear furanocoumarins psoralen, bergapten, xanthotoxin, isopimpinellin, imperatorin and their precursor umbelliferone.	Not detected	[83]
	Plantlets	MS with 2.0 mg/L NAA, 2.0 mg/L BA	Callus, suspension and hairy roots of *A. majus* containing umbelliferone were obtained.	Not detected	[84]
	Plantlets [84]	Not specified	The effect of various elicitors on the synthesis of coumarins (umbelliferone, marmesinin, scopoletin) in hairy roots of *A. majus* was shown.	Not detected	[85]
	Plantlets [84]	MS with various phytohormons	The effect of various elicitors on the synthesis of secondary metabolites (scopoletin, dehydrogeijerin) of in callus, cell suspension and hairy roots of *A. majus* by exposing them to elicitors.	Not detected	[86]
	Hairy root culture	MS with 2.5 mg/L NAA, 1.0 mg/L BA	Elicitation with ADR-4^®^ (electromagnetic treatment) induced also two times higher accumulation of bergapten.	Not detected	[87]
	Leafs	MS with 2 mg/L IAA *, 2 mg/L Kin *	Callus of *A. majus* were obtained. About 81% of calluses converted to shoot on medium with 50 mg/L glutamine and 40 mg/L adenine. Plantlets with shoot were transferred to 1/2 MS with different concentration of IBA and glutamine. Maximum rooting of 75.96% was observed on 1/2 MS supplemented with 0.2% of IBA and 100 mg/L glutamine.	Not detected	[88]
*Angelica archangelica* (L.) subsp. *archangelica*	Embryogenic cell line from seeds	Hormone-free, modified B5 *	Embryogenic cell line of *A. archangelica* was obtained, after 5 years it did not decrease capacity of embryo formation.	Not detected	[89]
	Plantlets from embryogenic cell line	-	14 coumarins were identified in the roots of *A. archangelica*, oxypeucedanin hydrate and oxypeucedanin being the main compounds.	Not detected	[90]
*Angelica gigas* Nakai	Young plants	Schenk and Hildebrandt media with 2.0 mg/L 2,4-D, 1.0 mg/L Kin	An immunostimulating polysaccharide was produced extracellularly by suspension cell culture of *A. gigas.*	Not detected	[91]
	Leaves and stems	Hormone-free MS	Hairy roots were induced from leaf and stem explants of *A. gigas*. Biomass growth and decursin production was faster in hairy roots than in wild type of *A. gigas*.	Decursin	[78]
	Stems, roots and hypocotyls In vitro	MS with NAA and 2,4-D, BA, GA * and TDZ *	The highest callus induction rate was obtained from in vitro germinated stem, root and hypocotyl on the MS medium with 1.0 mg/L NAA and 0.5 mg/L BA.	Not detected	[92]
*Angelica sinensis* (Oliv.) Diets.	Immature embryos	MS, B5, White	Study showed that embryogenic callus growth was more rapid on MS basal medium than on B5 or White medium. Suspension culture and somatic embryos were obtained from this callus.	Not detected	[93]
*Peucedanum japonicum* Thunb.	Root, leaf blade and petiole parts of seedlings In vitro	MS with 0.1–5.0 mg/L 2,4-D and 0.1–5.0 mg/L ABA *	Embryogenesis was induced in media MS with ABA from callus subculture (MS with 2,4-D). Four-month-old tissue culture plants derived from somatic embryos showed significantly of chlorogenic acid (10.5 mg/g dw).	Not detected	[94]
*Phlojodicarpus sibiricus* (Steph. e x Spreng.) K.-Pol.	Seeds, leaves, roots and hypocotyls In vitro	MS with c 1.0 mg/L 2,4-D and 0.5 mg/L BA	Callus was obtained from seeds, leaves, roots and hypocotyls in vitro. Dihydrosamidin, visnadin, khellactone derivatives were identified in callus cultures.	Dihydrosamidin, visnadin, khellactone derivatives	[68]
	Callus	MS with c 1.0 mg/L 2,4-D and 0.5 mg/L BA	Suspension culture was obtained from callus.	Not detected	[69]
*Seseli lehmannii* Degen	Callus and somatic embryos	MS with 1.0–2.0 mg/L BA and 0.5–2.0 mg/L NAA	Organogenesis of callus was induced in MS media with 1.0–2.0 mg/L BAP and 0.5–2.0 mg/L NAA.	Not detected	[95]
***Rutaceae***					
*Aegle marmelos* L.	Leaves	MS with 2.26 μM 2,4-D and 2.2 μM benzyl adenine	Callus was obtained from leaves on MS with 2,4-D and BA. Derived shoots from callus were rooted in vitro on MS medium supplemented with 12.3 μM indole-3-butyric acid.	Not detected	[96]
*Catharanthus roseus* Linn.	Leaves, roots, axillary buds, shoot tips	MS and B5 with various concentrations of 2,4-D, Kin, NAA	Callus culture was obtained. There was no significant difference in growing parameters between cultures in both types of media formulations on agar (MS or B5 salts). However, the alkaloid content was 2–3 times higher in suspension culture compared to agar medium in similar treatments.	Not detected	[97]
*Haplophyllum patavinum* (L.) G. Don fil.	Plantlets	B5 with 3 mg/L IAA and MS with 3 mg/L IAA	*H. patavinum* is not rich in coumarin compounds in vivo, but a selected cell strain exhibited in vitro coumarin biogenetic potentialities stronger than in vivo.	Not detected	[98]
*Ruta graveolens* L.	Whole plant	MS with 1.5 mg/L 2,4-D and 1.5 mg/L NAA	MS with 2, 4-D (1.5 mg/L) and NAA (1.5 mg/L) responded well by giving the maximum percentage of callus induction (97.22 ± 2.54). Coumarins were detected by GC-MS in biomass.	Not detected	[99]
***Fabaceae***					
*Psoralea corylifolia* L. (Buguchi)	Leaves and stems	MS with IAA	Rooting was induced in microshoots of *P. corylifolia*. Peroxidase activity increased considerably during root induction indicating a key role of peroxidase in rooting of *P. corylifolia* microshoots in vitro.	Not detected	[100]

* MS—Murashige-Skoog Medium; 2,4-D—2,4-dichlorophenoxyacetic acid; BA—6-benzylaminopurine; NAA—1-naphthaleneacetic acid; IAA—indole-3-acetic acid; Kin—kinetin; B5—Gamborg B5 medium; GA—gibberellic acid; TDZ—thidiazuron; ABA—abscisic acid.

## Data Availability

The data presented in this study are available in the article.

## Supplementary Materials

The following supporting information can be downloaded at: https://www.mdpi.com/article/10.3390/plants11223135/s1, Table S1: substituents of pyranocoumarins; Figure S1: structures of artificial pyranocoumarins; Table S2: the content of pyranocoumarins in plants. References [113,114,115,116,117,118,119,120,121,122,123,124,125,126,127,128,129,130,131,132,133,134,135,136,137,138,139,140,141,142,143,144,145,146,147,148,149,150,151,152,153,154,155,156,157] are cited in the Supplementary Materials.
