# Supplementary material for: Plant Pyranocoumarins: Description, Biosynthesis, Application
Source: Plants (Basel). 2022 Nov 16;11(22):3135. doi: 10.3390/plants11223135 (PMC9693251; doi:10.3390/plants11223135)
Supplement: Supplementary file 1 [file plants-11-03135-s001.zip › plants-1999048-supplementary.pdf]

Supplementary Materials:

Table S1. Substituents of pyranocoumarins.

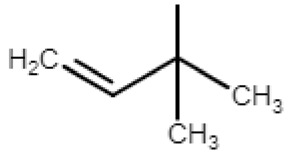	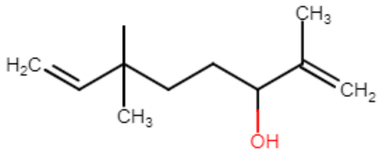	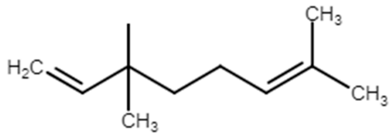
2-methylbut-3-en-2-yl	6-hydroxy-3,7-dimethylocta-1,7-dien-3-yl	3,7-dimethylocta-1,6-dien-3-yl
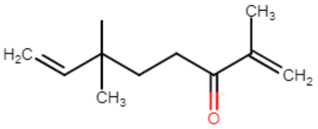	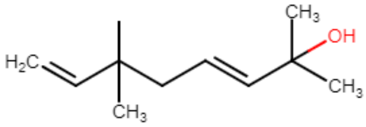	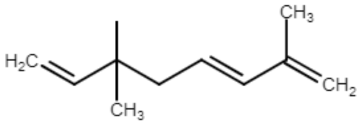
1-ethenyl-1,5-dimethyl-4-oxohex-5-en-1-yl	7-hydroxy-3,7-dimethylocta-1,5-dien-3-yl	3,7-dimethylocta-1,5,7-trien-3-yl
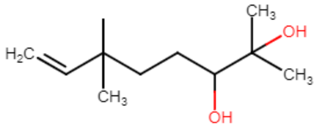	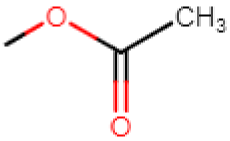	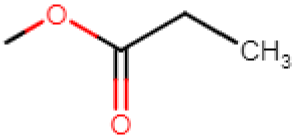
6,7-dihydroxy-3,7-dimethylocta-1-en-3-yl	acetyloxy	propionyloxy
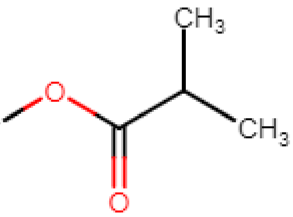	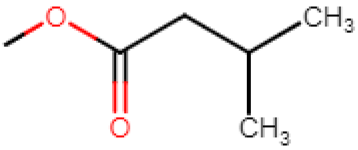	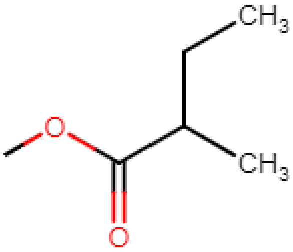
isobutyryloxy (isobutyroyloxy)	isovaleryloxy (isovaleroyloxy)	2-methylbutyroyloxy
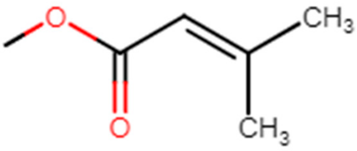	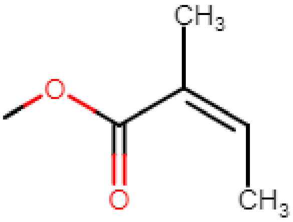	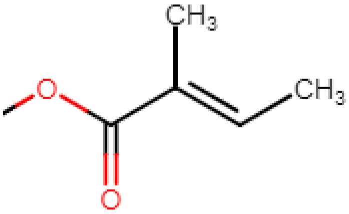
senecioyloxy	angeloyloxy	tigloyloxy
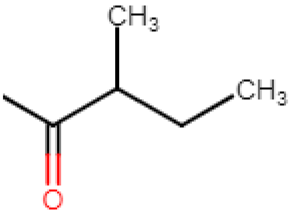	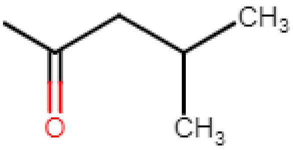	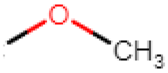
2-methyl-1-oxobutyl (2-methylbutyroyl?)	3-methyl-1-oxobutyl (isovaleroyl?)	methoxy

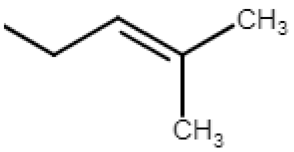	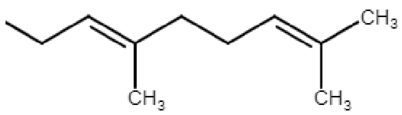	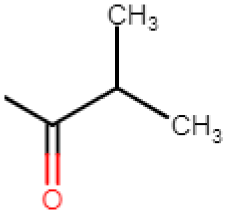
3-methyl-but-2-enyl	geranyl	isobutyryl
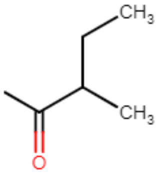	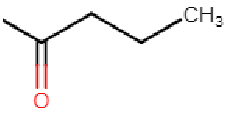	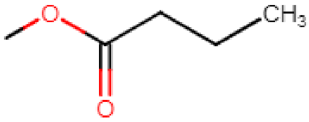
2-methylbutyroyl	butanal	butoxy
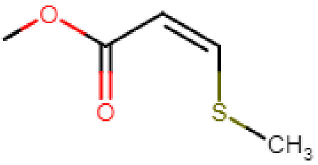	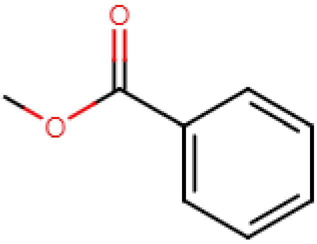	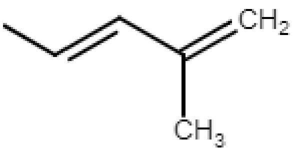
3-(Methylthio)acrylic acid	benzoyloxy	3-metyl-buta-1,3-dienyl
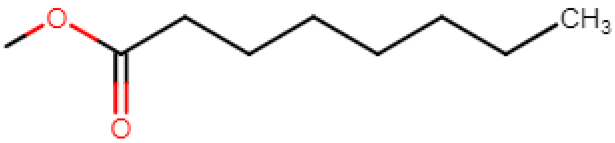		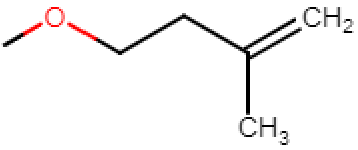
capryloyloxy		3-metyl-buta-3-enyloxy
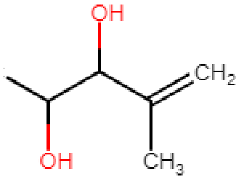	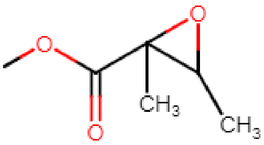	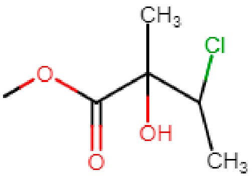
1,2-dihydroxy-3-metyl-but-3-enyl	2,3-dimethyloxirane-2-carboxylatyl	3-chloro-2-hydroxy-2-methylbutanoyl

Table S2. Structures of pyranocoumarins.

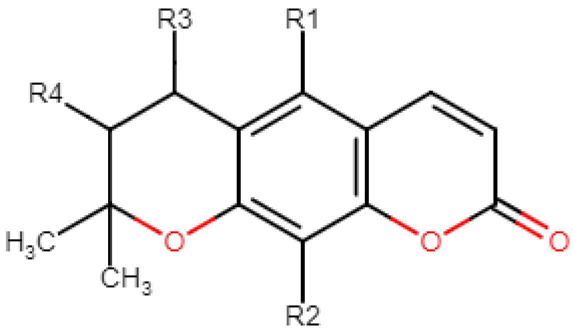

	R1	R2	R3	R4
Dihydroxanthyletin	H	H	H	H
(–)-3-(R)-Decursinol (Smirniol, Aegelinol)	H	H	H	R-OH
(+)-Decursinol ((–)-Smirniol)	H	H	H	S-OH
Decursidinol	H	H	OH	OH

(+)-trans-Decursidinol	H	H	R-OH	S-OH
(−)-*cis*-Decursidinol	H	H	S-OH	S-OH
Decursitin F	H	H	R-OH	S-acetoxy
4-Hydroxy Pd-C-III	H	H	R-OH	S- angeloyloxy
Pd-C-I	H	H	R-OH	S-senecioyloxy
Benzoyl aegelinol	H	H	H	benzoyloxy
Clausenin	OH	H	O	H
Clausenidin	OH	2,2-dimethylbut-3-enyl	O	H
Clauemarmarin A	OH	3,7-dimethylocta-1,5,7-trien-3-yl	H	H
Clauemarmarin B	OH	6,7-dihydroxy-3,7-dimethylocta-1-en-3-yl	H	H
Clauemarmarin C	OH	(R)-6-hydroxy-3,7-dimethylocta-1,7-dien-3-yl	H	H
Clauemarmarin D	OH	(S)-6-hydroxy-3,7-dimethylocta-1,7-dien-3-yl	H	H
5-hydroxy-8,8-dimethyl-10-(7-hydroxy-3,7-dimethylocta-1,5-dien-3-yl)pyranocoumarin	OH	7-hydroxy-3,7-dimethylocta-1,5-dien-3-yl	H	H
5-hydroxy-8,8-dimethyl-10-(3′,7′-dimethylocta-1′,6′-dien-3′-yl) pyranocoumarin	OH	3,7-dimethylocta-1,6-dien-3-yl	H	H
Clauemarmarin K	OH	(S/R)-1-ethenyl-1,5-dimethyl4-oxohex-5-en-1-yl	H	H
10-(7-Hydroxy-3,7-dimethylocta-1,5-dien-3-yl)-5-methoxy-8,8-dimethylpyranocoumarin	methoxy	7-hydroxy-3,7-dimethylocta-1,5-dien-3-yl	H	H
10-(3,7-Dimethylocta-1,6-dien-3-yl)-5-methoxy-8,8-dimethylpyranocoumarin	methoxy	3,7-dimethylocta-1,6-dien-3-yl	H	H
Dentatin	methoxy	2-methylbut-3-en-2-yl	H	H
Nordentain	OH	2-methylbut-3-en-2-yl		
Arnottianin	H	methoxy	H	OH
Decursin (Grandivitin)	H	H	H	S-senecioyloxy
Decursinol angelate	H	H	H	angeloyloxy
Grandivittin	H	H	H	senecioyloxy
Aegelinol benzoat	H	H	H	benzoyloxy
3′(R)-O-β-D-Glucopyranosyl-3′,4′ -dihydroxanthyletin	H	H	H	O-glucoside
Seseloside	H	OH	H	O-glucoside

(–)-methyl-Decursidinol	H	H	S- methoxy	R-OH
Peuarin	H	H	methoxy	angeloyloxy
4'-Methoxy Pd-C-I	H	H	R-methoxy	S-senecioyloxy
Pd-C-III	H	H	R-acetoxy	S-angeloyloxy
AD-I	H	H	R-isovaleryloxy	S-angeloyloxy
Pd-C-II	H	H	R-senecioyloxy	S-OH
Pd-C-IV	H	H	R-senecioyloxy	S-acetoxy
Decursidin	H	H	R-senecioyloxy	S-senecioyloxy
AD-II	H	H	R-senecioyloxy	S- angeloyloxy
3′(S)-Acetoxy-4′(R)-angeloyloxy-3′, 4′-dihydroxanthyletin	H	H	R-angeloyloxy	S-acetoxy
Decursitin D	H	H	R-angeloyloxy	S-OH
Pd-C-V	H	H	R-angeloyloxy	S-acetoxy
Decursitin C (Andelin)	H	H	R-angeloyloxy	S-senecioyloxy
Decursitin B	H	H	R-angeloyloxy	S-angeloyloxy
Xanthalin	H	H	angeloyloxy	tigloyloxy
Peuarenarine	H	H	2,3-dimethyloxirane-2-carboxylatyl	tigloyloxy
Peuarenine	H	H	2,3-dimethyloxirane-2-carboxylatyl	2,3-dimethyloxirane-2-carboxylatyl
Peuchlorin	H	H	3-chloro-2-hydroxy-2-methylbutanoyl	angeloyloxy
Peuchlorinin butyroyl isolhellaktone	H	H	3-chloro-2-hydroxy-2-methylbutanoyl	2,3-dimethyloxirane-2-carboxylatyl
Peuchloridin	H	H	3-chloro-2-hydroxy-2-methylbutanoyl	3-chloro-2-hydroxy-2-methylbutanoyl

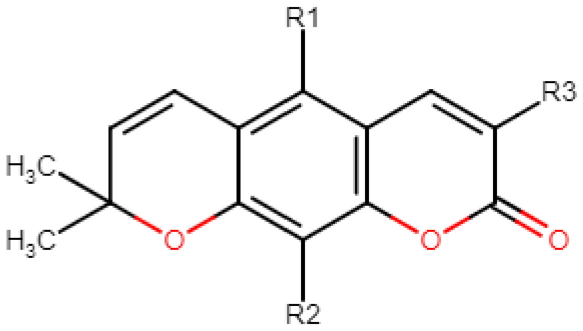

	R1	R2	R3
Xanthyletin	H	H	H
Luvangetin	H	methoxy	H
Xanthoxyletin	methoxy	H	H
Agasyllin	H	H	angeloyloxy
Xanthyletin 3-(1,1-dimetylbut-3-enyl)	H	H	1,1-dimetylbut-3-enyl
Xanthyletin 3-(3-metylbut-2-enyl)	H	H	3-metylbut-2-enyl
Xanthyletin 3-(1,1-dimetylbut-3-enyl)-8-(3-metylbut-2-enyl)	H	3-metylbut-2-enyl	1,1-dimetylbut-3-enyl
Nordentatin	OH	1,1-dimetylbut-3-enyl	H
Clausarin	OH	1,1-dimetylbut-3-enyl	1,1-dimetylbut-3-enyl
Trachyphyllin	OH	3-methyl-but-2-enyl	H
Poncitrin	methoxy	1,1-dimetylbut-3-enyl	H

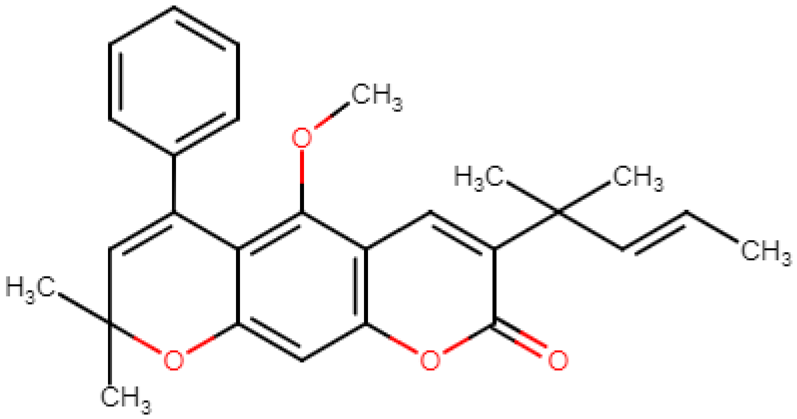

Phenyl derivative of pyranocoumarin (PDP)

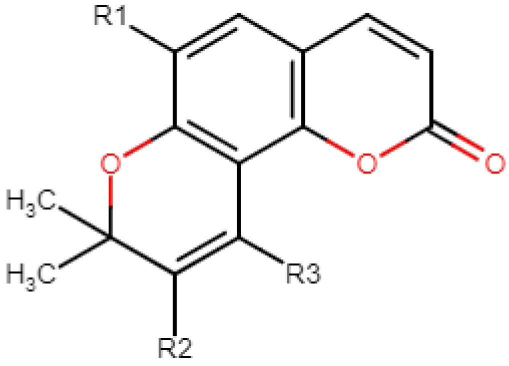

	R1	R2	R3
Seselin	H	H	H
Norbraylin	OH	H	H
Braylin	methoxy	H	H
Lomatin	H	OH	H
(–)-trans-Khellactone (cis-Khellactone; Visnagan)	H	OH	OH
trans-Khellactone / cis-Khellactone	H	R-OH	S-OH
(–)-cis-Ethylkhellactone	H	S-OH	S-ethoxy
(+)-trans-Ethylkhellactone	H	S-OH	R-ethoxy
Lomatin O-isovaleroyl ester	H	H	isovaleryloxy
Lomatin 2-methylbutyroyl ester	H	H	2-methylbutyroyloxy
Praeroside V	H	H	S-O- glucoside
Khellactone 4′-O-methyl ester	H	OH	methoxy
Khellactone 4′-O-acetyl ester	H	OH	acetyloxy
Qianhucoumarin C	H	S-OH	S-acetyloxy
Khellactone 4′-O-isobutyroyl ester	H	OH	isobutyryloxy
Khellactone 4′-O-isovaleroyl ester	H	OH	isovaleryloxy
Khellactone 4′-O- 2-methylbutyroyl ester	H	OH	2-methylbutyroyloxy
Turgeniifolin C	H	OH	senecioyloxy
Peujaponisinol B	H	S-OH	S-senecioyloxy
d-Laserpitin (Peujaponisinol B, Isolehmannidin)	H	OH	angeloyloxy
(+)-Laserpitin	H	S-OH	S-angeloyloxy

(±)-4′-Tigloylkhellactone	H	OH	tigloyloxy
Qianhucoumarin A	H	R-OH	R- tigloyloxy
Khellactone-4′-O-glucoside	H	OH	glucoside
Khellactone O-isobutyryl ester	H	OH / isobutyryloxy	isobutyryloxy / OH
Khellactone O-hexoside	H	OH / hexoside	hexoside / OH
Khellactone 3′-O-methyl ester	H	methoxy	OH
Qianhucoumarin B	H	S-acetyloxy	S-OH
3'-acetoxy-4'-metyl-3',4'-dihydroseselin	H	acetyloxy	metyloxy
Khellactone 3′,4′-di-O-acetyl ester	H	acetyloxy	acetyloxy
Qianhucoumarin D	H	S-acetyloxy	S-acetyloxy
3′(R)-acetoxy-4′(S)- propionyloxy - 3′,4′-dihydroseselin	H	R-acetyloxy	R-propionyloxy
Hyuganin D (Seravshanin, Isobocconin)	H	acetyloxy	isobutyryloxy
(3′S,4′R)-3′-acetyl-4′-isobutyrylkhellactone	H	S-acetyloxy	R-isobutyryloxy
Suksdorfin	H	acetyloxy	isovaleroyloxy
3′-acetyl-4′-isovalerylkhellactone	H	R-acetyloxy	R-isovaleroyloxy
Corymbocoumarin (+)-*cis*-3′acetoxy-4′-(2-methylbutyroyloxy)-3′,4′-dihydroseselin	H	acetyloxy	2-methylbutyroyloxy
Hyuganin C	H	acetyloxy	2-methyl-3-oxobutanoxy
Isosamidin	H	acetyloxy	senecioyloxy
(–)-trans-3′-Acetyl-4′-senecioylkhellactone	H	acetyloxy	senecioyloxy
Pteryxin	H	R-acetyloxy	R-angeloyloxy
Peucedanocoumarin II	H	S-acetyloxy	R-angeloyloxy
3′(R)-acetoxy-4′(S)-angeloyloxy-3′,4′-dihydroseselin	H	R-acetyloxy	S-angeloyloxy
Longshengensin A	H	S-acetyloxy	S-angeloyloxy
(±)-*cis*-3′-Acetyl-4′-tigloylkhellactone	H	acetyloxy	tigloyloxy
(+)-*trans*-3′-Acetyl-4′-tigloylkhellactone	H	acetyloxy	tigloyloxy
(3′R,4′S)-3′-acetyl-4′-tigloylkhellactone	H	R-acetyloxy	S-tigloyloxy
Peucedanocoumarin III	H	S-acetyloxy	R- tigloyloxy
Quanhucoumarin I	H	S-acetyloxy	S- tigloyloxy
Khellactone 3′-O- isobutyryl ester	H	isobutyryloxy	H
Campestrol	H	isobutyryloxy	OH
cis-3′-isobutyryl-4′-acetylkhellactone	H	R-isobutyryloxy	R-acetyloxy
3′.4′-di-*O*-isobutyryl-*cis*-khellactone	H	isobutyryloxy	isobutyryloxy
Lomatin isovalerate	H	isovaleryloxy	H
Hystrixarin (Turgeniifolin B)	H	isovaleryloxy	OH
3′-Isovaleryl-4′-keto-khellactone (Petracoumarin)	H	isovaleryloxy	O
Dihydrosamidin	H	isovaleryloxy	acetyloxy
Peucedanocoumarin I	H	S-isovaleryloxy	R-acetyloxy
Khellactone 3′-O-isovaleroyl-4′-O isobuturoyl ester	H	isovaleryloxy	isobutyryloxy
Khellactone 3′,4′-di-O-isovaleroyl	H	isovaleryloxy	isovaleroyloxy

3′(S), 4′(S)-diisovaleryloxy-3′,4′-dihydroseselin	H	S-isovaleryloxy	S-isovaleroyloxy
Khellactone isovaleroyl-2-methylbutyroyl ester	H	isovaleryloxy / 2-methylbutyroyloxy	2-methylbutyroyloxy / isovaleryloxy
Khellactone 3′-O-isovaleroyl-4′-O senecioyl ester	H	isovaleryloxy	senecioyloxy
cis-3′-isovaleryl-4′-senecioylkhellactone	H	S-isovaleryloxy	S-senecioyloxy
Khellactone 3′-O-isovaleroyl-4′-O angeloyl ester	H	isovaleryloxy	angeloyloxy
Visnadin	H	2-methylbutyroyloxy	acetyloxy
(–)-Visnadin	H	S-2-methylbutyroyloxy	S-acetyloxy
Khellactone 3′-O- 2-methylbutyroyl-4′ -O isobuturoyl ester	H	2-methylbutyroyloxy	isobutyryloxy
Khellactone 3′,4′-di-O-2-methylbutyroyl ester	H	2-methylbutyroyloxy	2-methylbutyroyloxy
Khellactone 3′-O-2-methylbutyroyl-4′-O senecioyl ester	H	2-methylbutyroyloxy	senecioyloxy
Khellactone 3′-O-isovaleroyl-4′-O angeloyl ester	H	2-methylbutyroyloxy	angeloyloxy
Khellactone 3′,4′-di-O-isoburyoyl ester	H	2-methylpropanoxy	isobutyryoyloxy
Buchtarmin	H	senecioyloxy	H
Isocampesol	H	senecioyloxy	OH
Peujaponisinol A	H	S-senecioyloxy	S-OH
3′(S)-senscioyloxy-4′ (S)-ethoxy-3′,4′-dihydroseselin	H	S-senecioyloxy	S-ethoxy
Samidin	H	senecioyloxy	acetyloxy
(+)-Samidin	H	S-senecioyloxy	S-acetyloxy
Khellactone 3′ -O-senecioyl-4′-O-isovaleroyl ester	H	senecioyloxy	isovaleroyloxy
Peujaponisin	H	S-senecioyloxy	S-isovaleroyloxy
Khellactone 3′-O-senecioyl-4′-O-2-methylbutyroyl ester	H	senecioyloxy	2-methylbutyroyloxy
cis-Khellactone disenecionate	H	senecioyloxy	senecioyloxy
3′(S),4′(S)-disenecioyloxy-3′,4′-dihydroseselin	H	S-senecioyloxy	S-senecioyloxy
Khellactone 3′-O-senecioyl-4′-O angeloyl ester	H	senecioyloxy	angeloyloxy
cis-3′-senecioyl-4′-angeloylkhellactone	H	R-senecioyloxy	R-angeloyloxy
Isocalypteryxin	H	senecioyloxy	tigloyloxy
Jatamansin (Selinidin, Xanthogalin)	H	angeloyloxy	H
Isolaserpetin	H	angeloyloxy	OH
(3′R,4′S)-3′-angeloylkhellactone	H	R-angeloyloxy	S-OH
Turgeniifolin A (Pd-Ib)	H	R-angeloyloxy	O
3'-angeloyloxy-4'-butoxy-3',4'-dihydroseselin	H	angeloyloxy	butoxy
(±)-Praeruptorin A (Pd-Ia)	H	angeloyloxy	acetyloxy
3′-angeloyl-4′-propionylkhellactone	H	angeloyloxy	propionyloxy
Qianhucoumarin J	H	S-angeloyloxy	S-propionyloxy

(-)-Praeruptorin A (Isopteryxin)	H	R-angeloyloxy	R-acetyloxy
(+)-Praeruptorin A	H	S-angeloyloxy	S-acetyloxy
Khellactone 3′-O-angeloyl-4′-O isobutyryl ester	H	angeloyloxy	isobutyryloxy
Praeruptorin E	H	angeloyloxy	isovaleroyloxy
(+)-Praeruptorin E (Qianhucoumarin H, Pd-III)	H	S- angeloyloxy	S-isovaleryloxy
Khellactone 3′-O- angeloyl-4′-O-2-methylbutyroyl ester	H	angeloyloxy	2-methylbutyroyloxy
Calypteryxin (Peuformosin)	H	angeloyloxy	senecioyloxy
(±)-Praeruptorin B (Anomalin)	H	angeloyloxy	angeloyloxy
(+)-Praeruptorin B (Pd-II, ((+)-Anomalin, Praeruptorin C)	H	R- angeloyloxy	R- angeloyloxy
(–)-Praeruptorin B (Praeruptorin D, Anomalin)	H	S- angeloyloxy	S- angeloyloxy
Khellactone 3′-O-tigloyl ester	H	tigloyloxy	OH
Qianhucoumarin E	H	R-tigloyloxy	O
(+)-trans-4′-Acetyl-3′-tigloylkhellactone	H	tigloyloxy	acetyloxy
Floroselin	H	tigloyloxy	3-(methylthio)acrylic acid
Campestrinol	H	geranyloxy	OH
Campestrinoside (Praeroside II)	H	O-glucoside	OH
Praeroside II	H	R-O-glucoside	R-OH
Praeroside IV	H	R-O-glucoside	H
Praeroside III	H	S-O-glucoside	R-OH
3'-Capryloyloxyxanthogalol (Octanoyllomatin)	H	capryloyloxy	H
Isofloroselin	H	3-(methylthio)acrylic acid	angeloyloxy

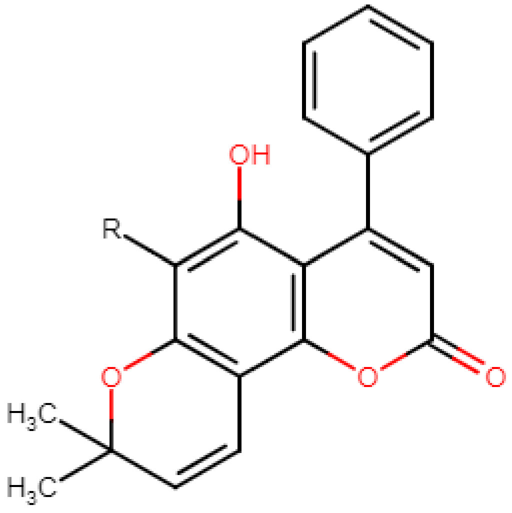

	R
mammea A/AA cyclo D	3-methyl-1-oxobutyl (isovaleroyl?)
mammea A/AD cyclo D	isobutyryl
mammea A/AB cyclo D	2-methylbutyroyl

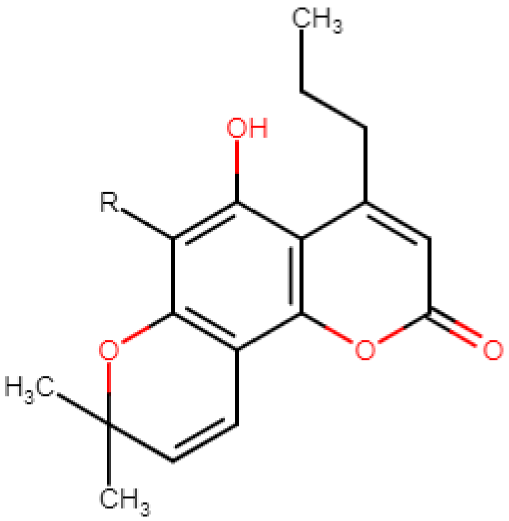

	R
mammea B/AB cyclo D	2-methyl-1-oxobutyl (2-methylbutyroyl?)
mammea B/AC cyclo D	butanal

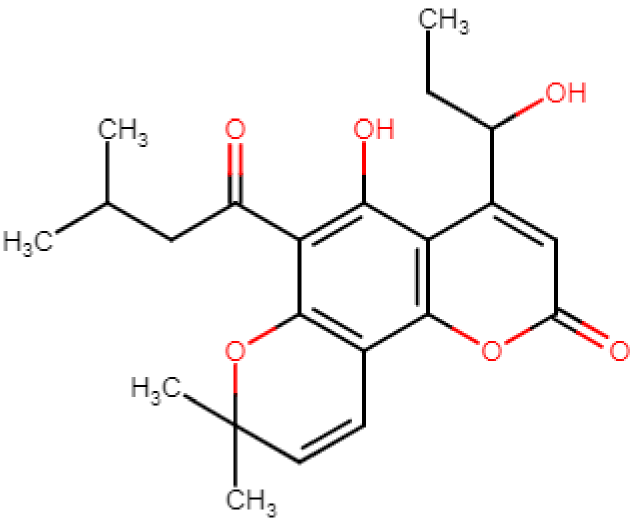

Deacetylmammea E/AA cyclo D

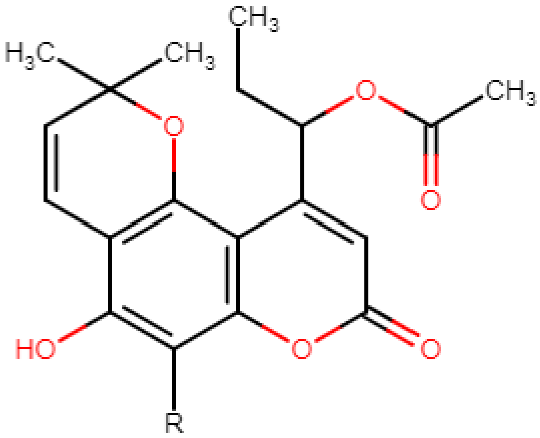

	R
Mammea E/BC cyclo D	butanal
Mammea E/BD cyclo D	isobutyryl

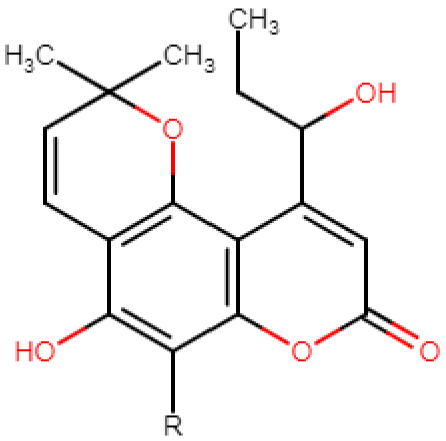

	R
Deacetylmammea E/BB cyclo D	2-methyl-1-oxobutyl (2-methylbutyroyl?)
Deacetylmammea E/BC cyclo D	butanal

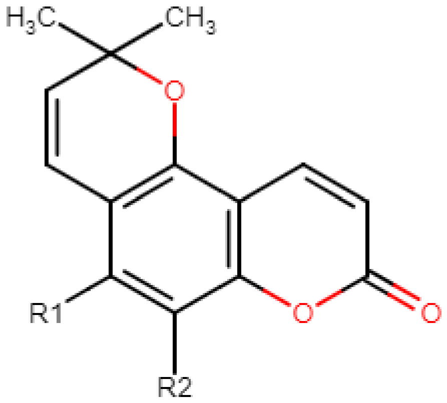

	R1	R2
Alloxanthoxyletin	methoxy	H
Avicennin	methoxy	3-metyl-buta-1,3-dienyl
Avicennol	methoxy	3-metyl-buta-3-enyloxy
Dipetaline	methoxy	3-methyl-but-2-enyl
c-Avicennol	methoxy	1,2-dihydroxy-3-metyl-but-3-enyl

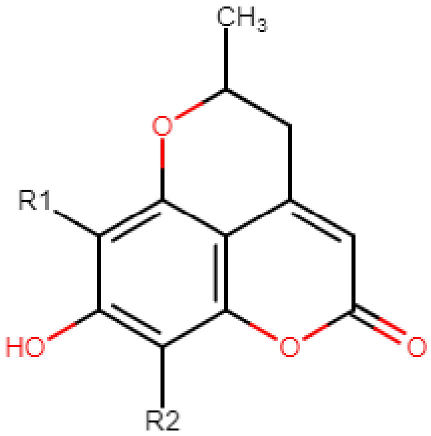

	R1	R2
8-hydroxy-5-methyl-7-(3-methyl-but-2-enyl)-9-(3-methyl-1-oxobutyl)-4,5-dihydropyrano[4,3,2-de]chromen-2-one	3-methyl-but-2-enyl	3-methyl-1-oxobutyl

8-hydroxy-5-methyl-7-(3-methyl-but-2-enyl)-9-(2-methyl-1-oxobutyl)-4,5-dihydropyrano[4,3,2-*de*]chromen-2-one	3-methyl-but-2-enyl	2-methyl-1-oxobutyl
8-hydroxy-5-methyl-7-(3,7-dimethyl-octa-2,6-dienyl)-9-(3-methyl-1-oxobutyl)-4,5-dihydropyrano[4,3,2-*de*]chromen-2-one	geranyl	3-methyl-1-oxobutyl
8-hydroxy-5-methyl-7-(3,7-dimethyl-octa-2,6-dienyl)-9-(2-methyl-1-oxobutyl)-4,5-dihydropyrano[4,3,2-*de*]chromen-2-one	geranyl	2-methyl-1-oxobutyl
Mammeasin C	geranyl	isobutyroyl
Mammeasin D	geranyl	butanal

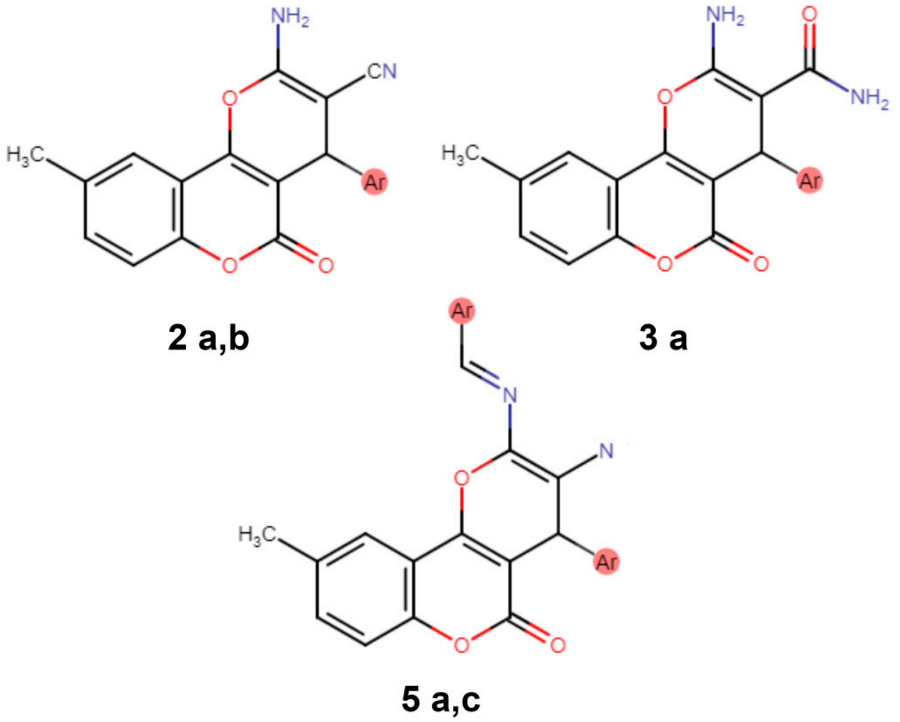

Figure S1. Structures of artificial pyranocoumarins [31]

Table S3. The content of pyranocoumarins in plants.

**Compounds**	**Species** *Umbelliferae (Apiaceae)*	**Plant parts** (yield, % dry mass) **Unknown**	**Whole plant**	**Aerial parts** **Flowers**	**Umbels**	**Stems/ Twigs**	**Leaves**	**Bark**	**Roots**	**Seeds**	**References**
Agasyllin (L*)	*Agasyllis latifolia* (M. Bieb.) Boiss.	+	?	?	?	?	?	–	?	?	[113]
Campestrinoside (Praeroside II) (Ad*)	*Ammi visnaga* (L.) Lam.	–	?	?	?	?	?	–	?	+	[114]
Visnadin (Ad)		–	?	?	+	?	?	–	?	0.46	[115-117]
Samidin (Ad)		–	?	?	+	?	?	–	+	0.08	[116-118]
Dihydrosamidin (Ad)		–	?	?	+	?	?	–	?	0.15	[116,117]
Decursin (Grandivitin) (L)		–	?	0.023				–	7.617	?	[119]
Seselin (Ad)		–	?	0.009				–	?	?	
(±)-Praeruptorin B (Anomalin) (Ad)		–	?	0.116				–	?	?	

(–)-Praeruptorin B (Praeruptorin D, Anomalin) (Ad)	*Angelica anomala* Ave-Lall.	+	?	?	?	?	?	–	?	?	[113]
Decursin (Grandivitin) (L)	*Angelica acutiloba*	+	?	?	?	?	?	–	?	?	[41]
Decursinol angelate (L)	(Siebold & Zucc.) Kitag.	+	?	?	?	?	?	–	?	?	
(–)-Praeruptorin B (Praeruptorin D, Anomalin) (Ad)	*Angelica adzharica* Pimenov	+	?	?	?	?	?	–	?	?	[113]
Decursitin C (Andelin) (L)	*Angelica decursiva* (Miq.)	+	?	?	?	?	?	–	?	?	[120,121]
(–)-3-(*R*)-Decursinol (Smirniol, Aegelinol) (L)	Franch. & Sav.	–	+	?	?	?	?	–	+	?	[121]
(+)-*trans*-Decursidinol (L)		–	+	?	?	?	?	–	?	?	
(–)-*cis*-Decursidinol (L)		–	?	?	?	?	?	–	+	?	
4-Hydroxy Pd-C-III (L)		–	+	?	?	?	?	–	?	?	
4'-Methoxy Pd-C-I (L)		–	+	?	?	?	?	–	?	?	
Pd-C-I (L)		–	+	?	?	?	?	–	?	?	
Pd-C-II (L)		–	+	?	?	?	?	–	?	?	
Pd-C-III (L)		–	+	?	?	?	?	–	?	?	
Pd-C-IV (L)		–	?	?	?	?	?	–	+	?	
Pd-C-V (L)		–	?	?	?	?	?	–	+	?	
Decursidin (L)		–	+	?	?	?	?	–	?	?	
Decursin (Grandivitin) (L)		–	?	?	?	?	?	–	+	?	
AD-I (L)		–	?	?	?	?	?	–	+	?	
AD-II (L)		–	?	?	?	?	?	–	+	?	
(–)-Methyl-Decursidinol (L)		–	?	?	?	?	?	–	+	?	
Decursitin B (L)		–	?	?	?	?	?	–	+	?	
Decursitin C (Andelin) (L)		–	?	?	?	?	?	–	+	?	
Decursitin D (L)		–	?	?	?	?	?	–	+	?	
Pteryxin (Ad)		–	+	?	?	?	?	–	?	?	
(3′*R*,4′*S*)-3′-acetyl-4′-Tigloylkhellactone (Ad)		–	+	?	?	?	?	–	?	?	
Jatamansin (Selinidin, Xanthogalin) (Ad)		–	?	?	?	?	?	–	+	?	
Peujaponisinol A (Ad)		–	?	?	?	?	?	–	+	?	
Peujaponisinol B (Ad)		–	?	?	?	?	?	–	+	?	
(–)-3-(*R*)-Decursinol (Smirniol, Aegelinol) (L)	*Angelica gigas* Nakai	–	?			+		–	?	?	[122]
(+)-Decursinol ((–)-Smirniol) (L)		–	?			+		–	?	?	
Agasyllin (L)		–	?			+		–	?	?	
Xanthyletin (L)		–	?			+		–	?	?	[123]
Decursin (Grandivitin) (L)		–	?	?	?	?	?	–	2.7-4.7	?	[124]
Decursinol angelate (L)		–	?	?	?	?	?	–	2.9-4.5	?	
Jatamansin (Selinidin, Xanthogalin) (Ad)	*Angelica purpurascens* (Ave-Lall.) Gilli	–	?	?	?	?	?	–	+	?	[125]
Xanthalin (L)		–	?	?	?	?	?	–	+	?	
Agasillin (L)		–	?	?	?	?	?	–	+	?	
(-)-Praeruptorin A (Isopteryxin) (Ad)	*Angelica sachalinensis* Maxim.	+	?	?	?	?	?	–	?	?	[113]
Decursin (Grandivitin) (L)	*Angelica sinensis*	+	?	?	?	?	?	–	?	?	[41]
Decursinol angelate (L)	(Oliv.) Diels	+	?	?	?	?	?	–	?	?	
(–)-Praeruptorin B (Praeruptorin D, Anomalin) (Ad)	*Angelica tatianae* Bordz.	+	?	?	?	?	?	–	?	?	[113]
Agasyllin (L)	*Eryngium campestre* L.	–	?	?	?	?	?	–	+	?	[126]
Decursin (Grandivitin) (L)		–	?	?	?	?	?	–	+	?	
Aegelinol benzoate (L)		–	?	?	?	?	?	–	+	?	
(–)-3-(*R*)-Decursinol (Smirniol, Aegelinol) (L)		–	?	?	?	?	?	–	+	?	
Grandivittin	*Ferulago campestris*	–	?	?	?	?	?	–	+	?	[33]

Agasyllin	(Besser) Grecescu	–	?	?	?	?	?	–	+	?	
Benzoyl aegelinol		–	?	?	?	?	?	–	+	?	
Grandivittin	*Ferulago macrocarpa* (Fenzl) Boiss.	–	?	?	?	?	?	–	?	+	[127]
Buchtarmin (Ad)	*Ferulopsis hystrix* (Bunge) Pimenov (*Phlojodicarpus turczaninovii* Sipliv.)	+	?	?	?	?	?	–	?	?	[113]
3′,4′-di-*O*-Isobutyryl-*cis*-khellactone (Ad)	*Glehnia litoralis* F. Schmidt ex Miq.	–	+	?	?	?	?	–	?	?	[128]
Samidin (Ad)		–	+	?	?	?	?	–	?	?	
Dihydrosamidin (Ad)		–	+	?	?	?	?	–	?	?	
cis-Khellactone disenecionate		–	+	?	?	?	?	–	?	?	
Khellactone 3′-*O*-isovaleroyl-4′-*O* senecioyl ester (Ad)		–	+	?	?	?	?	–	?	?	
Khellactone 3′,4′-di-*O*-isovaleroyl (Ad)		–	+	?	?	?	?	–	?	?	
Jatamansin (Selinidin, Xanthogalin) (Ad)	*Ligusticum lucidum* Mill. subsp. *cuneifolium*	–	?			+			?	?	[28]
(±)-Praeruptorin A (Pd-Ia) (Ad)	(Guss.) Tammaro	–	?			+			?	?	
Visnadin (Ad)		–	?			+			?	?	
(±)-Praeruptorin B (Anomalin) (Ad)	*Musineon divaricatum* (Pursh) Raf	–	+	?	?	?	?	–	?	?	[129]
d-Laserpitin (Peujaponisinol B, Isolehmannidin) (Ad)		–	+	?	?	?	?	–	?	?	
Isolaserpetin (Ad)		–	+	?	?	?	?	–	?	?	
Turgeniifolin C (Ad)		–	+	?	?	?	?	–	?	?	
Isocampesol (Ad)		–	+	?	?	?	?	–	?	?	
Pteryxin (Ad)		–	+	?	?	?	?	–	?	?	
Isosamidin (Ad)		–	+	?	?	?	?	–	?	?	
Suksdorfin (Ad)		–	+	?	?	?	?	–	?	?	
Khellactone 4′-*O*-isovaleroyl ester (Ad)		–	+	?	?	?	?	–	?	?	
Praeruptorin E (Ad)		–	+	?	?	?	?	–	?	?	
Khellactone-3′-*O*-isobutyryl ester (Ad)		–	+	?	?	?	?	–	?	?	
Khellactone 3′-*O*-angeloyl-4′-*O* isobutyryl ester (Ad)		–	+	?	?	?	?	–	?	?	
Khellactone 4′-*O*- 2-methylbutyroyl ester (Ad)		–	+	?	?	?	?	–	?	?	
Khellactone 3′-*O*-tigloyl ester (Ad)		–	+	?	?	?	?	–	?	?	
Calypteryxin (Peuformosin) (Ad)		–	+	?	?	?	?	–	?	?	
Khellactone 3′-*O*-senecioyl-4′-*O* angeloyl ester (Ad)		–	+	?	?	?	?	–	?	?	
Khellactone (Ad)		–	+	?	?	?	?	–	?	?	
Jatamansin (Selinidin, Xanthogalin) (Ad)		–	+	?	?	?	?	–	?	?	
(–)-Methyl-Decursidinol (L)	*Peucedanum arenarium* Waldst. & Kit.	–	?	?	?	?	?	–	+	?	[130]
Decursitin B (L)		–	?	?	?	?	?	–	+	?	
Peuarenine (L)		–	?	?	?	?	?	–	+	?	
Peuarin (L)		–	?	?	?	?	?	–	+	?	
Peuarenarine (L)		–	?	?	?	?	?	–	+	?	
Peuchlorin (L)		–	?	?	?	?	?	–	+	?	
Peuchlorinin butyroyl isolhellaktone (L)		–	?	?	?	?	?	–	+	?	
Peuchloridin (L)		–	?	?	?	?	?	–	+	?	

Decursidin (L)	*Peucedanum decursivum* (Miq.) Maxim	–	?	?	?	?	?	–	+	?	[131]
Pd-C-IV (L)		–	?	?	?	?	?	–	+	?	
Pd-C-V (L)		–	?	?	?	?	?	–	+	?	
Decursitin C (Andelin) (L)		–	?	?	?	?	?	–	+	?	[130]
Dihydroxanthyletin (L)		–	?	?	?	?	?	–	+	?	[130]
(+)-*trans*-Decursidinol (L)		–	?	?	?	?	?	–	+	?	
3′(*S*)-Acetoxy-4′(*R*)-angeloyloxy-3′, 4′-dihydroxanthyletin (L)		–	?	?	?	?	?	–	+	?	
Decursitin B (L)		–	?	?	?	?	?	–	+	?	
Decursitin (L)		–	?	?	?	?	?	–	+	?	
Decursitin D (L)		–	?	?	?	?	?	–	+	?	
Decursitin F (L)		–	?	?	?	?	?	–	+	?	
Pd-C-III (L)		–	?	?	?	?	?	–	+	?	
(–)-Praeruptorin B (Praeruptorin D, Anomalin) (Ad)	*Peucedanum delavayi* Franch.	–	?	?	?	?	?	–	+	?	[130]
3′(*R*)-*O*-β-D-Glucopyranosyl-3′,4′ -dihydroxanthyletin (L)	*Peucedanum dissolutum* (Diels) H. Wolff	–	?	?	?	?	?	–	+	?	[130]
(–)-Praeruptorin A (Isopteryxin) (Ad)		–	?	?	?	?	?	–	+	?	
(–)-*cis*-Khellactone (Ad)	*Peucedanum formosanum* Hayata	–	?	?	?	?	?	–	+	?	[130]
(–)-Praeruptorin B (Praeruptorin D, Anomalin) (Ad)		–	?	?	?	?	?	–	+	?	
Isosamidin (Ad)		–	?	?	?	?	?	–	+	?	
Corymbocoumarin (Ad)		–	?	?	?	?	?	–	+	?	
Dihydroxanthyletin (L)	*Peucedanum harry-smithii var. subglabrum* (Shan & M.L. Sheh) Shan & M.L. Sheh	–	?	?	?	?	?	–	+	?	[130]
Calypteryxin (Peuformosin) (Ad)		–	?	?	?	?	?	–	+	?	
(±)-Praeruptorin A (Pd-Ia) (Ad)		–	?	?	?	?	?	–	+	?	
(–)-Praeruptorin B (Praeruptorin D, Anomalin) (Ad)		–	?	?	?	?	?	–	+	?	
Longshengensin A (Ad)	*Peucedanum longshengense* R. H. Shan & M. L. Sheh	–	?	?	?	?	?	–	+	?	[130]
(+)-*trans*-Khellactone (Ad)	*Peucedanum japonicum* Thunb.	–	?			+		–	?	?	[132]
(+)-*trans*-4′-Acetyl-3′-tigloylkhellactone (Ad)		–	?			+		–	?	?	
(+)-Praeruptorin A (Ad)		–	?			+		–	?	?	
3′(*S*),′(*S*)-diisovaleryloxy-3′,4′-dihydroseselin (Ad)		–	+			+		–	+	?	[130,133]
3′(*S*),4′(*S*)-disenecioyloxy-3′,4′-dihydroseselin (Ad)		–	?	?	?	?	?	–	+	?	[133]
(+)-Samidin (Ad)		–	?	?	?	?	?	–	+	?	
Peujaponisin (Ad)		–	?	?	?	?	?	–	+	?	
(–)-Visnadin (Ad)		–	?	?	?	?	?	–	+	?	
(–)-Praeruptorin B (Praeruptorin D, Anomalin) (Ad)		–	?	?	?	?	?	–	+	?	
(–)-*cis*-Khellactone (Ad)		–	?	?	?	?	?	–	+	?	
(–)-*trans*-Khellactone (Ad)		–	?	?	?	?	?	–	+	?	
(–)-*cis*-Ethylkhellactone (Ad)		–	?	?	?	?	?	–	+	?	
(+)-*trans*-Ethylkhellactone (Ad)		–	?	?	?	?	?	–	+	?	
3′(*S*)-Senscioyloxy-4′ (*S*)-ethoxy-3′,4′-dihydroseselin (Ad)		–	?	?	?	?	?	–	+	?	
Peucedanocoumarin I (Ad)		–	?	?	?	?	?	–	+	?	
Peujaponisinol A (Ad)		–	?	?	?	?	?	–	+	?	[134]
Peujaponisinol B (Ad)		–	?	?	?	?	?	–	+	?	
(±)-*cis*-3′-Acetyl-4′-Tigloylkhellactone (Ad)		–	?			+		–	?	?	[130]
(–)-*trans*-3′-Acetyl-4′-		–	?			+		–	?	?	

senecioylkhellactone (Ad)											
cis-3′-isovaleryl-4′ -senecioylkhellactone (Ad)		–	?	+				–	?	?	
Peucedanocoumarin III (Ad)		–	?	?	?	?	?	–	+	?	
(±)-Praeruptorin A (Pd-Ia) (Ad)		–	?	?	?	?	?	–	+	?	
Pteryxin (Ad)		–	?	?	?	?	?	–	+	?	
Jatamansin (Selinidin, Xanthogalin) (Ad)		–	?	?	?	?	?	–	+	?	
(±)-4′-Tigloylkhellactone (Ad)		–	?	?	?	?	?	–	+	?	
Praeroside II (Ad)		–	?	?	?	?	+	–	?	?	
Praeroside III (Ad)		–	?	?	?	?	+	–	?	?	
Praeroside IV (Ad)		–	?	?	?	?	+	–	?	?	
Praeroside V (Ad)		–	?	?	?	?	+	–	?	?	
Peucedanocoumarin III (Ad)	*Peucedanum medium var. gracile* Dunn ex Shan et Sheh	–	?	?	?	?	?	–	+	?	[130]
Peucedanocoumarin III (Ad)	*Peucedanum ostruthium* (L.) W.D.J. Koch—Masterwort	–	?	?	?	?	?	–	+	?	[130]
(±)-Praeruptorin A (Pd-Ia) (Ad)	*Peucedanum praeruptorum* Dunn	–	?	?	?	?	?	–	+	?	[135]
(±)-Praeruptorin B (Anomalin) (Ad)		–	?	?	?	?	?	–	+	?	
Jatamansin (Selinidin, Xanthogalin) (Ad)		–	?	?	?	?	?	–	+	?	[130]
3′(*S*),′(*S*)-diisovaleryloxy-3′,4′-dihydroseselin (Ad)		–	?	?	?	?	?	–	+	?	
Calypteryxin (Peuformosin) (Ad)		–	?	?	?	?	?	–	+	?	
(+)-Praeruptorin A (Ad)		–	?	?	?	?	?	–	+	?	[136]
(+)-Praeruptorin B (Pd-II, ((+)-Anomalin, Praeruptorin C) (Ad)		–	?	?	?	?	?	–	+	?	
(–)-Praeruptorin A (Isopteryxin) (Ad)		–	?	?	?	?	?	–	+	?	
(–)-Praeruptorin B (Praeruptorin D, Anomalin) (Ad)		–	?	?	?	?	?	–	+	?	
(+)-Praeruptorin E (Pd-III) (Ad)		–	?	?	?	?	?	–	+	?	
Turgeniifolin A (Pd-Ib) (Ad)		–	?	?	?	?	?	–	+	?	
Quanhucoumarin I (Ad)		–	?	?	?	?	?	–	+	?	
(3′*R*,4′*S*)-3′-acetyl-4′-Tigloylkhellactone (Ad)		–	?	?	?	?	?	–	+	?	
Pteryxin (Ad)		–	?	?	?	?	?	–	+	?	
(3′*S*,4′*R*)-3′-acetyl-4′-Isobutyrylkhellactone (Ad)		–	?	?	?	?	?	–	+	?	
Peucedanocoumarin I (Ad)		–	?	?	?	?	?	–	+	?	
Peucedanocoumarin II (Ad)		–	?	?	?	?	?	–	+	?	
Peucedanocoumarin III (Ad)		–	?	?	?	?	?	–	+	?	
(+)-Praeruptorin E (Qianhucoumarin H, Pd-III) (Ad)		–	?	?	?	?	?	–	+	?	
(3′*R*,4′*S*)-3′-angeloylkhellactone (Ad)		–	?	?	?	?	?	–	+	?	
Qianhucoumarin A (Ad)		–	?	?	?	?	?	–	+	?	
Qianhucoumarin B (Ad)		–	?	?	?	?	?	–	+	?	
Qianhucoumarin C (Ad)		–	?	?	?	?	?	–	+	?	
Qianhucoumarin D (Ad)		–	?	?	?	?	?	–	+	?	
Qianhucoumarin E (Ad)		–	?	?	?	?	?	–	+	?	
(+)-Laserpitin (Ad)		–	?	?	?	?	?	–	+	?	
Qianhucoumarin J (Ad)		–	?	?	?	?	?	–	+	?	
cis-Khellactone disenecionate (Ad)		–	?	?	?	?	?	–	+	?	

Hyuganin D (Seravshanin, Isobocconin) (Ad)		–	?	?	?	?	?	–	+	?	
trans-Khellactone (Ad)		–	?	?	?	?	?	–	+	?	
cis-Khellactone (Ad)		–	?	?	?	?	?	–	+	?	
3′-Acetyl-4′-isovalerylkhellactone (Ad)		–	?	?	?	?	?	–	+	?	
cis-3′-Senecioyl-4′-angeloylkhellactone (Ad)		–	?	?	?	?	?	–	+	?	
Khellactone 3′,4′-di-*O*-isovaleroyl (Ad)		–	?	?	?	?	?	–	+	?	
3′-Isovaleryl-4′-keto-khellactone (Petracoumarin) (Ad)		–	?	?	?	?	?	–	+	?	
3′-Angeloyl-4′-Propionylkhellactone (Ad)		–	?	?	?	?	?	–	+	?	
Praeroside II (Ad)		–	?	?	?	?	?	–	+	?	
Praeroside III (Ad)		–	?	?	?	?	?	–	+	?	
Praeroside IV (Ad)		–	?	?	?	?	?	–	+	?	
Praeroside V (Ad)		–	?	?	?	?	?	–	+	?	
cis-3′-Isovaleryl-4′-senecioylkhellactone (Ad)		–	?	?	?	?	?	–	+	?	
cis-3′-Isobutyryl-4′-acetylkhellactone (Ad)		–	?	?	?	?	?	–	+	?	
Decursin (Grandivitin) (L)	*Peucedanum terebinthaceum var.*	–	?	?	?	?	?	–	+	?	[130]
Pteryxin (Ad)	*deltoideum* (Makino ex K.Yabe) Makino	–	?	?	?	?	?	–	+	?	
Turgeniifolin A (Pd-Ib) (Ad)	*Peucedanum turgeniifolium*	–	+	?	?	?	?	–	?	?	[130]
Hystrixarin (Turgeniifolin B) (Ad)	H. Wolff	–	+	?	?	?	?	–	?	?	
Turgeniifolin C (Ad)		–	+	?	?	?	?	–	?	?	
3′(*S*)-Acetoxy-4′(*R*)-angeloyloxy-3′, 4′-dihydroxanthyletin (L)	*Peucedanum wawrii* (Wolff) Su By	–	?	?	?	?	?	–	+	?	[137]
3′(*R*)-Acetoxy-4′(*S*)-angeloyloxy-3′,4′-dihydroseselin (Ad)		–	?	?	?	?	?	–	+	?	
(+)-Decursinol ((–)-Smirniol) (L)	*Peucedanum wulongense* R.	–	?	?	?	?	?	–	+	?	[130]
(+)-*trans*-Khellactone (Ad)	H. Shan & M. L. Sheh	–	?	?	?	?	?	–	+	?	
3′(*S*),4′(*S*)-Disenecioyloxy-3′,4′-dihydroseselin (Ad)		–	?	?	?	?	?	–	+	?	
(–)-Praeruptorin B (Praeruptorin D, Anomalin) (Ad)		–	?	?	?	?	?	–	+	?	
Praeruptorin E (Ad)	*Peucedanum zenkeri* L.	–	?	?	?	?	?	–	?	+	[130]
Dihydrosamidin (Ad)	*Phlojodicarpus villosus* (Turcz. ex Fisch. et C.A.	–	?	?	?	?	?	–	2	?	[138]
Decursin (Grandivitin) (L)	Mey.) Ledeb	+	?	?	?	?	?	–	?	?	[113]
(–)-Decursinol (Smirniol, Aegelinol) (L)		+	?	?	?	?	?	–	?	?	
Visnadin (Ad)		+	?	?	?	?	?	–	?	?	
Khellactone (Visnagan) (Ad)	*Phlojodicarpus sibiricus*	–	?	?	?	?	?	–	+	?	[51,139]
Khellactone 4′-*O*-methyl ester (Ad)	(Steph. ex Spreng.) K.-Pol.	–	?	?	?	+		–	?	+	
Khellactone 4′-*O*-isovaleroyl ester / khellactone 4′-*O*-2-methylbutyroyl ester (Ad)		–	?	?	?	?	?	–	+	?	
Khellactone 4′-O-acetyl ester (Ad)		–	?	?	?	+		–	?	+	
Lomatin *O*-isovaleroyl ester / lomatin 2-methylbutyroyl ester (Ad)		–	?	?	?	?	?	–	+	?	

Khellactone 3′,4′-di-*O*-acetyl ester (Ad)	–	?	+	?	+		–	0.252	+
Khellactone 4′-*O*-isobutyroyl ester (Ad)	–	?	?	?	?	?	–	+	?
d-Laserpitin (Peujaponisinol B, Isolehmannidin) (Ad)	–	?	?	?	0.1251		–	0.47	0.214
Hyuganin D (Seravshanin, Isobocconin) (Ad)	–	?	+	?	+		–	0.149	0.01
Pteryxin (Ad)	–	?	?	?	?	?	–	+	?
Dihydrosamidin (Ad)	–	?	+	?	1.085		–	8.014	1.228
Suksdorfin (Ad)	–	?	?	?	?	?	–	+	?
Hyuganin C (Ad)	–	?	?	?	+		–	0.053	+
cis-Khellactone disenecionate (Ad)	–	?	?	?	?	?	–	+	?
(–)-Praeruptorin B (Praeruptorin D, Anomalin) (Ad)	–	?	+	?	?	?	–	+	?
Khellactone 3′-*O*-isovaleroyl-4′-*O* senecioyl ester / Khellactone 3′-*O*-2-methylbutyroyl-4′-*O* senecioyl ester / Khellactone 3′-*O*-isovaleroyl-4′-*O* angeloyl ester / Khellactone 3′-*O*-isovaleroyl-4′-*O* angeloyl ester (Ad)	–	?	?	?	?	?	–	+	?
Khellactone 3′-*O*-isovaleroyl-4′-*O* isobuturoyl ester / Khellactone 3′-*O*- 2-methylbutyroyl-4′ -*O* isobuturoyl ester (Ad)	–	?	?	?	?	?	–	+	?
Khellactone 3′ -*O*-senecioyl-4′-*O*-isovaleroyl ester / Khellactone 3′-*O*-senecioyl-4′-*O*-2-methylbutyroyl ester / Praeruptorin E / Khellactone 3′-*O*- angeloyl-4′-*O*-2-methylbutyroyl ester (Ad)	–	?	?	?	?	?	–	+	?
Khellactone 3′,4′-di-*O*-isovaleroyl / Khellactone 3′,4′-di-*O*-2-methylbutyroyl ester / Khellactone isovaleroyl-2-methylbutyroyl ester (Ad)	–	?	?	?	?	?	–	+	?
Khellactone 3′,4′-di-*O*-isoburyoyl ester (Ad)	–	?	?	?	?	?	–	+	?
Khellactone-4′-*O*-glucoside	–	?	?	?	+		–	+	?
Campestrinoside (Praeroside II) (Ad)	–	?	+	?	1.059		–	0.139	0.046
Khellactone *O*-isobutyryl Ester (Ad)	–	?	?	?	+		–	?	?
Khellactone *O*-hexoside (Ad)	–	?	?	?	+		–	?	?
Khellactone 3′-*O*-methyl Ester (Ad)	–	?	?	?	+		–	?	?
3′.4′-Di-*O*-isobutyryl-*cis*-khellactone (Ad)	–	?	+	?	?	?	–	?	?

3′(*R*)-Acetoxy-4′(*S*)- propionyloxy -3′,4′-dihydroseselin (Ad)	*Prionosciadium watsonii* J.M. Coult. & Rose	+	?	?	?	?	?	–	?	?	[140]
Khellactone 4′-O-isobutyroyl ester (Ad)		+	?	?	?	?	?	–	?	?	
Isolaserpetin (Ad)		+	?	?	?	?	?	–	?	?	
Hyuganin D (Seravshanin, Isobocconin) (Ad)		+	?	?	?	?	?	–	?	?	
Khellactone 3′,4′-di-*O*-acetyl ester (Ad)		+	?	?	?	?	?	–	?	?	
(–)-*trans*-Khellactone (*cis*-Khellactone; Visnagan) (Ad)		+	?	?	?	?	?	–	?	?	
Khellactone 4′-*O*-methyl ester (Ad)		+	?	?	?	?	?	–	?	?	
Jatamansin (Selinidin, Xanthogalin) (Ad)		+	?	?	?	?	?	–	?	?	
Khellactone 3′-*O*- isobutyryl ester (Ad)		+	?	?	?	?	?	–	?	?	
Lomatin (Ad)		+	?	?	?	?	?	–	?	?	
(+)-Decursinol ((–)-Smirniol)	*Saposhnikovia divaricata* (Turcz.) Schischk.	–	?	?	?	?	?	–	+	?	[43]
(–)-Praeruptorin B (Praeruptorin D, Anomalin)		–	?	?	?	?	?	–	+	?	
Calypteryxin (Peuformosin) (Ad)	*Seseli arenarium* M. Bieb.	–	?	?	?	?	?	–	+	?	[141]
Isosamidin (Ad)	(*Seseli campestre* Bess.)	–	?	?	?	?	?	–	+	?	
Campestrinoside (Praeroside II) (Ad)		–	?	?	?	?	?	–	+	?	[142]
(-)-*trans*-Khellactone (Ad)		–	?	?	?	?	?	–	+	?	
Campestrol (Ad)		–	?	?	?	?	?	–	+	?	
Campestrinol (Ad)		–	?	?	?	?	?	–	+	?	
Isocampesol (Ad)		–	?	?	?	?	?	–	+	?	
Isocalypteryxin (Ad)		–	?	?	?	?	?	–	+	?	
Isolaserpetin (Ad)		–	?	?	?	?	?	–	+	?	
3'-Acetoxy-4'-metyl-3',4'-dihydroseselin (Ad)		–	?	?	?	?	?	–	+	?	
(-)-Praeruptorin B (Ad)		+	?	?	?	?	?	–	?	?	[113]
(-)-Praeruptorin B (Ad)	*Seseli asperulum* (Trautv.) Schischk.	+	?	?	?	?	?	–	?	?	[113]
Pteryxin (Ad)	*Seseli condensatum* (L.) Rchb. f. (*Libanotis condensata* ssp. *arctica* (Rupr.) V.G. Sergienko)	+	?	?	?	?	?	–	?	?	[143]
Isofloroselin (Ad)	*Seseli coronatum* Ledeb.	+	?	?	?	?	?	–	?	?	[144]
(-)-Praeruptorin B (Ad)		+	?	?	?	?	?	–	?	?	[113]
cis-Khellactone (Ad)	*Seseli devenyense* Simonk.	–	?	?	?	?	?	–	?	+	[60]
d-Laserpitin (Peujaponisinol B, Isolehmannidin) (Ad)		–	?	?	?	?	?	–	?	+	
Isolaserpetin (Ad)		–	?	?	?	?	?	–	?	+	
3'-Capryloyloxyxanthogalol (Octanoyllomatin) (Ad)		–	?	?	?	?	?	–	?	+	
(-)-Praeruptorin B (Ad)	*Seseli dichotomum* Pall. ex M.Bieb.	+	?	?	?	?	?	–	?	?	[113]
(-)-Praeruptorin B (Ad)	*Seseli incanum* (Stephan ex Willd.) B. Fedtsch.	+	?	?	?	?	?	–	?	?	[113]
cis-Khellactone disenecionate (Ad)		+	?	?	?	?	?	–	?	?	
(-)-Praeruptorin B (Ad)	*Seseli iliense* Lipsky	+	?	?	?	?	?	–	?	?	[113]
Pteryxin (Ad)	*Seseli jomuticum* Schischk.	+	?	?	?	?	?	–	?	?	[143]
Decursin (Grandivitin) (L)	*Seseli grandivittatum*	+	?	?	?	?	?	–	?	?	[113]

(-)-3-(*R*)-Decursinol (L)	(Sommier & Levier)	+	?	?	?	?	?	–	?	?	
Decursinol angelate (L)	Schischk.	+	?	?	?	?	?	–	?	?	
(–)-Praeruptorin B (Praeruptorin D, Anomalin) (Ad)		+	?	?	?	?	?	–	?	?	
Corymbocoumarin (Ad)	*Seseli gummiferum* Pall. ex Sm. subsp. *corymbosum* (Boiss. & Heldr.) P.H. Davis	+	?	?	?	?	?	–	?	?	[26]
Pteryxin (Ad)	*Seseli mucronatum* (Schrenk) Pimenov & Sdobnina	+	?	?	?	?	?	–	?	?	[143]
Pteryxin (Ad)	*Seseli nemorosum* Pimenov	+	?	?	?	?	?	–	?	?	[143]
d-Laserpitin (Peujaponisinol B, Isolehmannidin) (Ad)	*Seseli lehmannianum* (Bunge) Boiss. (*Libanotis*	+	?	?	?	?	?	–	?	?	[113]
cis-Khellactone (Ad)	*lehmanniana* Bunge)	+	?	?	?	?	?	–	?	?	
Lomatin (Ad)		+	?	?	?	?	?	–	?	?	
3'-Capryloyloxyxanthogalol (Octanoyllomatin) (Ad)		+	?	?	?	?	?	–	?	?	
3'-Angeloyloxy-4'-butoxy-3',4'-dihydroseselin (Ad)		+	?	?	?	?	?	–	?	?	
Pteryxin (Ad)		+	?	?	?	?	?	–	?	?	[143]
3′-Isovaleryl-4′-keto-khellactone (Petracoumarin) (Ad)	*Seseli petraeum* M. Bieb.	–	?			+			–	?	[61]
3'-Capryloyloxyxanthogalol (Octanoyllomatin) (Ad)		–	?			+			–	?	
Jatamansin (Selinidin, Xanthogalin) (Ad)		–	?			+			–	?	
(–)-Praeruptorin B (Praeruptorin D, Anomalin) (Ad)		–	?			+			–	?	
Khellactone 3′-*O*- isobutyryl ester (Ad)		–	?			+			–	?	
Praeruptorin E (Ad)		–	?			+			–	?	
Khellactone 3′-*O*-isovaleroyl-4′-*O* angeloyl ester		–	?			+			–	?	
Khellactone 3′-*O*-senecioyl-4′-*O* angeloyl ester		–	?			+			–	?	
Samidin		–	?			+			–	?	
Peujaponisinol B		–	?			+			–	?	
Peujaponisinol A		–	?			+			–	?	
cis-Khellactone		–	?			+			–	?	
Seseloside (L)	*Seseli peucedanoides* (M. Bieb.) Koso-Pol.	+	?	?	?	?	?	–	?	?	[145]
(–)-Praeruptorin B (Praeruptorin D, Anomalin) (Ad)	*Seseli sessiliflorum* Schrenk (*Seseli tschuense* E. Nikit.)	+	?	?	?	?	?	–	?	?	[113]
(–)-Praeruptorin B (Praeruptorin D, Anomalin) (Ad)		+	?	?	?	?	?	–	?	?	
Floroselin (Ad)		+	?	?	?	?	?	–	?	?	[143]
Khellactone 3′-*O*-isovaleroyl-4′-*O* angeloyl ester (Ad)	*Seseli talassicum* Pimenov & Sdobnina	+	?	?	?	?	?	–	?	?	[113]
Praeruptorin C (Ad)	*Seseli tenuisectum* Regel &	+	?	?	?	?	?	–	?	?	[113]
(-)-Praeruptorin B (Ad)	Schmalh.	+	?	?	?	?	?	–	?	?	
Agasyllin (L)		+	?	?	?	?	?	–	?	?	
cis-Khellactone (Ad)		+	?	?	?	?	?	–	?	?	
trans-Khellactone (Ad)		+	?	?	?	?	?	–	?	?	
Pteryxin (Ad)	*Seseli valentinae* Popov	+	?	?	?	?	?	–	?	?	[143]
(–)-3-(*R*)-Decursinol (Smirniol,	*Smyrniopsis aucheri* Boiss.	+	?	?	?	?	?	–	?	?	[143]

Aegelinol) (L)											
(–)-3-(*R*)-Decursinol (Smirniol, Aegelinol) (L)	*Zosima absinthifolia* (Vent) Link	+	?	?	?	?	?	–	?	?	[146]
Agasyllin (L)		+	?	?	?	?	?	–	?	?	
Agasyllin (L)	*Zosima korovinii* Pimenov	+	?	?	?	?	?	–	?	?	[147]
	Rutaceae										
Decursinol (L)	*Aegle marmelos* L.	+	?	?	?	?	?	?	?	?	[148]
Xanthyletin (L)	*Afreagle paniculata* (Schumach. & Thonn.) Engl.	+	?	?	?	?	?	?	?	?	[148]
Xanthoxyletin (L)		+	?	?	?	?	?	?	?	?	
Seselin (Ad)	*Atalantia buxifolia* (Poir.) Oliv. ex Benth. (*Severinia buxifolia* Ten.)	+	?	?	?	?	?	?	?	?	[148]
Xanthyletin 3-(3-metylbut-2-enyl) (L)	*Atalantia simplicifolia* (Roxb.) Engl. (*Amyris simplicifolia* Roxb.)	–	?	?	?	?	+	?	?	?	[148]
Xanthyletin (L)	*Atalantia monophylla* (Roxb.) A. DC.	–	?	?	?	?	?	?	+	?	[148]
Xanthyletin (L)	*Boenninghausenia albiflora* (Hook.) Rchb. ex Meisn.	+	?	?	?	?	?	?	?	?	[148]
Xanthyletin 3-(1,1-dimetylbut-3-enyl) (L)		+	?	?	?	?	?	?	?	?	
Xanthyletin (L)	*Citrus aurantiifolia* (Christm.) Swingle, (*Citrus acida* Roxb.)	+	?	?	?	?	?	?	?	?	[148]
Xanthyletin (L)	*Citrus aurantium* L.	+	?	?	?	?	?	?	?	?	[148]
Seselin (Ad)		+	?	?	?	?	?	?	?	?	
Poncitrin (L)		+	?	?	?	?	?	?	?	?	
Hystrixarin (Turgeniifolin B) (Ad)	*Citrus hystrix* D.C.	–	?	?	?	?	?	?	+	?	[149]
Decursidinol (L)		–	?	?	?	?	?	?	+	?	
Xanthyletin (L)		–	?	?	?	?	?	?	+	?	
Seselin (Ad)		–	?	?	?	?	?	?	+	?	
Seselin (Ad)	*Citrus limetta* Risso	+	?	?	?	?	?	?	?	?	[148]
Xanthyletin (L)	*Citrus limettioides* Tanaka	+	?	?	?	?	?	?	?	?	[148]
Xanthyletin (L)	*Citrus limon* (L.) Osbeck	+	?	?	?	?	?	?	+	?	[148,150]
Xanthoxyletin (L)		–	?	?	?	?	?	?	+	?	[150]
Xanthyletin (L)	*Citrus medica* L.	–	?	?	?	?	?	?	+	?	[148]
Nordentatin (L)		–	?	?	?	?	?	?	+	?	
Xanthyletin (L)	*Citrus nobilis v. sunkii* Lour.	+	?	?	?	?	?	?	?	?	[148]
Xanthyletin (L)	*Citrus paradisi* Macfad.	+	?	?	?	?	?	?	?	?	[148]
Seselin		+	?	?	?	?	?	?	?	?	
Xanthyletin (L)	*Citrus sinensis* (L.) Osbeck.	+	?	?	?	+	+	?	?	?	[148,150]
Xanthoxyletin (L)		–	?	?	?	+	?	?	?	?	[150]
Xanthyletin (L)	*Citrus tankan* Hayata	+	?	?	?	?	?	?	?	?	[148]
Xanthyletin (L)	*Chloroxylon swietenia* DC.	+	?	?	?	?	?	?	?	?	[148]
Xanthoxyletin (L)		+	?	?	?	?	?	?	?	?	
Luvangetin (L)		+	?	?	?	?	?	?	?	?	
Alloxanthoxyletin (Au)		+	?	?	?	?	?	?	?	?	
Xanthyletin (L)	*Choisya arizonica* Standl.	–	?	?	?	+	?	?	?	?	[148]
Xanthyletin (L)	*Choisya mollis* Standl.	–	?	?	?	+	?	?	?	?	[148]
Xanthyletin (L)	*Choisya ternata* Kunth.	+	?	?	?	?	?	?	?	?	[148]
Xanthoxyletin (L)	*Clausena anisata* (Willd.) Hook.f. ex Benth.	–	?	?	?	?	?	?	+	?	[148]
Xanthyletin 3-(1,1-dimetylbut-3-		–	?	?	?	?	?	?	+	?	

enyl) (L)											
Nordentatin (L)	*Clausena dentata* (Willd.)	–	?	?	?	?	?	?	+	?	[148]
Poncitrin (L)	Roem.	–	?	?	?	?	?	?	+	?	
Clauemarmarin A (L)	*Clausena emarginata* C. C.	–	?	?	?	+	?	?	?	?	[59]
Clauemarmarin B (L)	Huang	–	?	?	?	+	?	?	?	?	
Clauemarmarin C (L)		–	?	?	?	+	?	?	?	?	
Clauemarmarin D (L)		–	?	?	?	+	?	?	?	?	
5-hydroxy-8,8-dimethyl-1*O*-(7-hydroxy-3,7-imethylocta-1,5-dien-3-l)pyranocoumarin (L)		–	?	?	?	+	?	?	?	?	
5-hydroxy-8,8-dimethyl-1*O*-(3′,7′-dimethylocta-1′,6′-dien-3′-yl) pyranocoumarin (L)		–	?	?	?	+	?	?	?	?	
Clauemarmarin K		–	?	?	?	+	?	?	?	?	[27]
10-(7-Hydroxy-3,7-dimethylocta-1,5-dien-3-yl)-5-methoxy-8,8-dimethylpyranocoumarin		–	?	?	?	+	?	?	?	?	
10-(3,7-Dimethylocta-1,6-dien-3-yl)-5-methoxy-8,8-dimethylpyranocoumarin		–	?	?	?	+	?	?	?	?	
Dentatin		–	?	?	?	+	?	?	?	?	
Nordentain		–	?	?	?	+	?	?	?	?	
Clausenin (L)	*Clausena excavata*	+	?	?	?	?	?	?	?	?	[148]
Clausenidin (L)	Burm.f.	+	?	?	?	?	?	?	?	?	[44,151]
Nordentatin (L)		–	?	?	?	?	?	?	+	?	[44]
Clausarin (L)		–	?	?	?	?	?	?	+	?	
Xanthoxyletin (L)		–	?	?	?	?	?	?	+	?	
Clausenin (L)	*Clausena heptaphylla*	–	?	?	?	?	?	?	+	?	[152]
Poncitrin (L)	(Roxb.) Wight & Arn.	+	?	?	?	?	?	?	?	?	[148]
Clausenidin (L)		–	?	?	?	?	?	?	+	?	[152]
Poncitrin (L)	*Clausena pentaphylla* Lam. ex DC.	+	?	?	?	?	?	?	?	?	[148]
Clausarin (L)		+	?	?	?	?	?	?	?	?	
Seselin (Ad)	*Flindersia bennettii* (F.Muell.) ex C.Moore (*Flindersia benniata*)	+	?	?	?	?	?	?	?	?	[148]
Braylin (Ad)	*Flindersia brayleyana* F.Muell.	+	?	?	?	?	?	?	?	?	[148]
Seselin (Ad)	*Flindersia ifflana* F.Muell.	–	?	?	?	?	?	+	?	?	
Xanthyletin (L)	*Flindersia pimenteliana*	–	?	?	?	?	?	+	?	?	
Seselin (Ad)	F.Muell	–	?	?	?	?	?	+	?	?	
Xanthyletin (L)	*Glycosmis cyanocarpa* (Blume) Spreng.	+	?	?	?	?	?	?	?	?	
Xanthoxyletin (L)	*Halfordia kendack* (Montrouz.) Guillaumin (*Halfordia scleroxyla* F.Muell.)	–	?	?	?	?	?	+	?	?	
Seselin (Ad)	*Haplophyllum cappadocicum* Spach	–	+	?	?	?	?	?	?	?	[153]
Seselin (Ad)	*Haplophyllum dubium* Korovin	+	?	?	?	?	?	?	?	?	[144]
Xanthyletin (L)	*Haplophyllum dshungaricum*	–	+	?	?	?	?	?	?	?	[153]
Seselin (Ad)	Rubtzov	+	?	?	?	?	?	?	?	?	[144]
Lomatin isovalerate (Ad)	*Haplophyllum kowalenskyi*	+	?	?	?	?	?	?	?	?	[114]

	Stschegl.										
Xanthyletin (L)	*Haplophyllum multicaule*	+	?	?	?	?	?	?	?	?	[144]
Seselin (Ad)	Vved.	+	?	?	?	?	?	?	?	?	[148]
Seselin (Ad)	*Haplophyllum schelkovnikovii* Grossh.	–	?			+			?	?	[153]
Lomatin isovalerate (Ad)	*Haplophyllum tenue* Boiss.	+	?	?	?	?	?	?	?	?	[114]
Seselin (Ad)	*Haplophyllum thesioides* (Fisch. ex DC.) G.Don	–	?			+			?	?	[154]
Luvangetin (L)	*Hesperathusa crenulata* Roem.	–	?	?	?	?	+	?	?	?	[148]
Xanthyletin (L)	*Hortia arborea* Engl.	–	?	?	?	?	?	?	+	?	[148]
Xanthyletin 3-(1,1-dimetylbut-3-enyl) (L)		–	?	?	?	?	?	?	+	?	[148]
Xanthoxyletin (L)	*Luvunga eleutherandra* Dalz.	–	?	?	?	+	?	?	?	?	[148]
Xanthoxyletin (L)	*Luvunga scandens* (Roxb.)	–	?	?	?	?	?	?	?	+	[148]
Luvangetin (L)	Buch. Ham.	–	?	?	?	?	?	?	?	+	[148]
Xanthoxyletin (L)	*Melicope mantellii* Buch.	–	?	?	?	?	?	+	?	?	[148]
Xanthoxyletin (L)	*Melicope ternata* Forst.	–	?	?	?	?	?	+	?	?	[148]
Braylin (Ad)	*Pitavia punctata* Mol.	–	?	?	?	+	+	?	?	?	[148]
Seselin (Ad)	*Phebalium squamulosum* Vent.(*Phebalium argenteum*)	–	?	?	?	?	+	?	?	?	[148]
Avicennin (Au*)	*Philotheca coccinea* (C.A.Gardner) Paul G.Wilson (*Eriostemon coccineus* C.A.Gardner)	–	?	?	?	+	?	?	?	?	[148]
Xanthoxyletin (L)	*Philotheca obovalis* (Cunn.) Paul G.Wilson (*Eriostemon obovalis* A.Cunn.)	+	?	?	?	?	?	?	?	?	[148]
Xanthoxyletin (L)	*Philotheca trachyphylla*	+	?	?	?	?	?	?	?	?	[148]
Trachyphyllin (L)	(F.Muell.) Paul G.Wilson (*Eriostemon trachyphyllus* F.Muell.)	+	?	?	?	?	?	?	?	?	
Poncitrin (L)	*Poncirus trifoliata* (L.) Raf.	+	?	?	?	?	?	?	?	?	[148]
Seselin (Ad)		+	?	?	?	?	?	?	?	?	
Xanthoxyletin (L)	*Ruta graveolens* L.	+	?	?	?	?	?	?	?	?	[148]
Xanthyletin 3-(1,1-dimetylbut-3-enyl)-8-(3-metylbut-2-enyl) (L)		+	?	?	?	?	?	?	?	?	
Xanthoxyletin (L)	*Ruta microcapra* Svent.	–	?	?	?	?	+	?	?	?	[148]
Luvangetin (L)		–	?	?	?	?	+	?	?	?	
Xanthoxyletin (L)	*Ruta oreojasme* Webb.	+	?	?	?	?	?	?	?	?	[148]
Luvangetin (L)		+	?	?	?	?	?	?	?	?	
Seselin (Ad)		+	?	?	?	?	?	?	?	?	
Xanthoxyletin (L)	*Ruta pinnata* L. fil.	+	?	?	?	?	?	?	?	?	[148]
Luvangetin (L)		+	?	?	?	?	?	?	?	?	
Seselin (Ad)		+	?	?	?	?	?	?	?	?	
Seselin (Ad)	*Scimmia repens* Nakai	–	?	?	?	+	?	?	?	?	[148]
Jatamansin (Selinidin, Xanthogalin) (Ad)	*Xanthogallum purpurascens* Lallem.	–	?	?	?	?	?	?	+	?	[113,155]
Lomatin (Ad)		+	?	?	?	?	?	?	?	?	[144]
Xanthyletin (L)	*Zanthoxylum ailanthoides* Siebold & Zucc.	+	?	?	?	?	?	?	?	?	[148]
Xanthyletin (L)	*Zanthoxylum americanum*	–	?	?	?	?	?	?	+	?	[148]
Xanthoxyletin (L)	Mill.	–	?	?	?	?	?	?	+	?	
Arnottianin	*Zanthoxylum arnottianum*	+	?	?	?	?	?	?	?	?	[148]

Xanthyletin (L)	Maxim.	+	?	?	?	?	?	?	?	?	
Avicennin (Au)	*Zanthoxylum avicennae*	–	?	?	?	?	?	?	+	?	[148]
Avicennol (Au)	Lam.	–	?	?	?	?	?	?	+	?	
Luvangetin (L)	*Zanthoxylum asiaticum*	+	?	?	?	?	?	?	?	?	[148]
Norbraylin (Ad)	(L.) Appelhans, Groppo & J.Wen (*Toddalia aculeata* Pers.)	+	?	?	?	?	?	?	?	?	
Xanthoxyletin (L)	*Zanthoxylum dipetalum* H.	–	?	?	?	?	?	?	+	?	[148]
Dipetaline (Au)	Mann	–	?	?	?	?	?	?	+	?	
Avicennol (Au)		–	?	?	?	?	?	?	+	?	
Xanthoxyletin (L)	*Zanthoxylum elephantiasis*	+	?	?	?	?	?	?	?	?	[148]
Avicennin (Au)	Macfad.	+	?	?	?	?	?	?	?	?	
Avicennol (Au)		+	?	?	?	?	?	?	?	?	
c-Avicennol (Au)		+	?	?	?	?	?	?	?	?	
Xanthyletin (L)	*Zanthoxylum faurei* Ohwi	–	?	?	?	?	?	?	+	?	[148]
Xanthyletin (L)	*Zanthoxylum pluviatile* Hartley	–	?	?	?	?	+	?	?	?	[148]
	Calophyllaceae										
Mammea A/AD cyclo D (Ad)	*Mammea siamensis* T. Anders.	–	?	+	?	+	?	?	?	?	[18,67]
Mammea A/AA cyclo D (Ad)		–	?	?	?	+	?	?	?	?	
Mammea A/AB cyclo D (Ad)		–	?	+	?	+	?	?	?	?	
8-hydroxy-5-methyl-7-(3-methyl-but-2-enyl)-9-(3-methyl-1-oxobutyl)-4,5-dihydropyrano[4,3,2-de]chromen-2-one (C)		–	?	+	?	+	?	?	?	?	
8-hydroxy-5-methyl-7-(3,7-dimethyl-octa-2,6-dienyl)-9-(3-methyl-1-oxobutyl)-4,5-dihydropyrano[4,3,2-*de*]chromen-2-one (C)		–	?	?	?	+	?	?	?	?	
8-hydroxy-5-methyl-7-(3-methyl-but-2-enyl)-9-(2-methyl-1-oxobutyl)-4,5-dihydropyrano[4,3,2-*de*]chromen-2-one (C)		–	?	+	?	+	?	?	?	?	[18,67]
8-hydroxy-5-methyl-7-(3,7-dimethyl-octa-2,6-dienyl)-9-(2-methyl-1-oxobutyl)-4,5-dihydropyrano[4,3,2-*de*]chromen-2-one (C)		–	?	?	?	+	?	?	?	?	[18]
Mammeasin C (C)		–	?	+	?	?	?	?	?	?	[67]
Mammeasin D (C)		–	?	+	?	?	?	?	?	?	
Mammea B/AB cyclo D (Ad)		–	?	+	?	?	?	?	?	?	
Mammea B/AC cyclo D (Ad)		–	?	+	?	?	?	?	?	?	
Mammea E/BC cyclo D (Au)		–	?	+	?	?	?	?	?	?	
Mammea E/BD cyclo D (Au)		–	?	+	?	?	?	?	?	?	
Mammea E/AA cyclo D (Ad)		–	?	+	?	?	?	?	?	?	
Mammea E/BB cyclo D (Au)		–	?	+	?	?	?	?	?	?	
Mammea E/BC cyclo D (Au)		–	?	+	?	?	?	?	?	?	
	Cornaceae										

Decursitin C (Andelin) (L)	*Camptotheca acuminata* Decne.	–	?	?	?	?	?	?	+	?	[156]
	Fabaceae										
Phenyl derivative of pyranocoumarin (PDP) (L)	*Psoralea corylifolia* L.	–	?	?	?	?	?	?	?	+	[21]
	Ranunculaceae										
Luvangetin (L)	*Eranthis longistipitata* Regel.	–	+	?	?	?	?	–	?	?	[157]

*Ad –angular 7,8-pyranocoumarin; L – linear 6,7-pyranocoumarin; Au – angular 5,6-pyranocoumarin; C – condensed 4,5-pyranocoumarin.
